# Decision Support Algorithm for Selecting an Antivirus Mask over COVID-19 Pandemic under Spherical Normal Fuzzy Environment

**DOI:** 10.3390/ijerph17103407

**Published:** 2020-05-13

**Authors:** Zaoli Yang, Xin Li, Harish Garg, Meng Qi

**Affiliations:** 1 College of Economics and Management, Beijing University of Technology, Beijing 100124, China; yangzaoli@bjut.edu.cn (Z.Y.); xinl@emails.bjut.edu.cn (X.L.); 2 School of Mathematics, Thapar Institute of Engineering and Technology, Deemed University, Patiala Pin 147004, India; harish.garg@thapar.edu

**Keywords:** COVID-19, antivirus mask selection, spherical normal fuzzy set, Bonferroni mean operator, multi-criteria decision-making

## Abstract

With the rapid outbreak of COVID-19, most people are facing antivirus mask shortages. Therefore, it is necessary to reasonably select antivirus masks and optimize the use of them for everyone. However, the uncertainty of the effects of COVID-19 and limits of human cognition add to the difficulty for decision makers to perfectly realize the purpose. To maximize the utility of the antivirus mask, we proposed a decision support algorithm based on the novel concept of the spherical normal fuzzy (SpNoF) set. In it, firstly, we analyzed the new score and accuracy function, improved operational rules, and their properties. Then, in line with these operations, we developed the SpNoF Bonferroni mean operator and the weighted Bonferroni mean operator, some properties of which are also examined. Furthermore, we established a multi-criteria decision-making method, based on the proposed operators, with SpNoF information. Finally, a numerical example on antivirus mask selection over the COVID-19 pandemic was given to verify the practicability of the proposed method, which the sensitive and comparative analysis was based on and was conducted to demonstrate the availability and superiority of our method.

## 1. Introduction

Since the discovery of COVID-19 in Wuhan, China in December 2019, it has spread rapidly in China. Moreover, more than 190 countries and regions around the world have seen the infection quickly spread [1]. As a large family of viruses, coronaviruses are known to cause colds and more serious diseases such as MERS and SARS [2], among which COVID-19 is a new coronavirus strain that has never been found in humans before. Common signs of a person infected with COVID-19 include respiratory symptoms, fever, cough, shortness of breath, and dyspnea. In more severe cases, infection can result in pneumonia, severe acute respiratory syndrome, kidney failure, and even death [3]. As far as we know, the primary transmission route of COVID-19 is respiratory droplets and contact [4]. Under the COVID-19 epidemic, masks have become essential items for medical staff and ordinary people to work and travel. However, the global demand for masks and other personal protective equipment is now 100 times that of the usual level and the price is 20 times that of ordinary times. Even worse, people’s inappropriate and excessive use of personal protective equipment will further exacerbate this mask shortage situation to make it persist for a long time. Hence, optimizing the use of antivirus masks according to disparate people is the efficacious basic measure to deal with the mask shortage and COVID-19 diffusion.

Admittedly, with this terrible virus, everyone is eager to get the best mask, but different groups of people should have dissimilar specific needs. Especially under the situation of COVID-19 spreading and a mask shortage, it is everyone’s basic duty and obligation to resist COVID-19 without excessive protection. Therefore, for different groups of people, the rational choice and utilization of masks has pivotal practical significance. Obviously, the best masks do not mean the most expensive ones. For most people, it is not necessary to use masks with the same standard as those used by front-line medical staff. The suitability of an antivirus mask is not only related to the protective effect of the mask itself, but also to the tightness (the leakage rate) of the combination of the mask and the human face. In addition, under the severe epidemic situation and the lack of antivirus masks, the choice of masks is also relevant to factors such as reusability and quality of raw materials. Therefore, for the vast majority of people, under the severe epidemic situation, it is essential to consider multiple factors when choosing a mask to optimize the allocation of medical resources. However, people’s limited knowledge and the uncertainty of the COVID-19 expansion increases the difficulty and complexity for decision-making about selecting a reasonable antivirus mask. To cope with this challenge, this paper proposed a multi-criteria decision-making (MCDM) method for selecting an antivirus mask under spherical normal fuzzy using the Bonferroni Mean operator.

The study yields a number of contributions as follows:

(1) A novel concept of the Spherical normal fuzzy (SpNoFS) set is defined, between which the new score, the accuracy function, and some improved operational rules are established.
(2) Some new information aggregation operators based on operational rules of SpNoFS, including the Spherical normal fuzzy Bonferroni mean (SpNoFBM) operator and the Spherical normal fuzzy weighted Bonferroni mean (SpNoFGBM) operator, are proposed.
(3) A new MCDM method for selecting an antivirus mask over the COVID-19 pandemic in light of the SpNoFBM operator and the SpNoFGBM operator is constructed.

The rest of this paper is arranged as follows: Section 2 reviews literature on healthcare and medical decision-making problems and MCDM methods based on fuzzy theories. Section 3 provides an overview of the normal fuzzy number and the Spherical fuzzy number. Section 4 presents the concept of the Spherical normal fuzzy number (SpNoFN) and defines its operations, score, and accuracy function, as well as sorting rules between SpNoFNs. Section 5 develops both the SpNoFNBM operator and the SpNoFGBM operator. Section 6 constructs a MCDM method based on those aggregation operators. Section 7 gives a numerical example of the antivirus mask, selecting an antivirus mask over the COVID-19 pandemic to verify the availability of the proposed method. Section 8 summarizes some general conclusions.

## 2. Literature Review

### 2.1. The Healthcare and Medical Decision Making Problems Based on the Fuzzy MCDM Method

With the advantage of being able to deal fuzzy and uncertain information, fuzzy MCDM methods are widely used for decision-making in medical and health fields. For medical diagnosis, Akram et al. [5] developed a novel bipolar fuzzy MCDM method to evaluate patients’ health status and identify the influencing factors. Hashmi et al. [6] developed a new concept of m-polar neutrosophic topology-based MCDM for the diagnosis of medical diagnosis problems. Zhou et al. [7] proposed a new divergence measure of Pythagorean fuzzy sets based on the Dempster-Shafer evidence theory to diagnose disease. For health assessment, Yucesan and Gul [8] proposed a fuzzy MCDM framework using the Pythagorean fuzzy analytic hierarchy process (AHP) and technique for ordering preference by similarity to ideal solution (TOPSIS) to evaluate hospital service quality. In addition, Lee et al. [9] proposed a hybrid fuzzy MCDM approach, combining AHP and similarity to ideal solution methods to evaluate Taiwan’s medical device manufacturers. Tadic et al. evaluated the suppliers of medical devices using a fuzzy TOPSIS method. Furthermore, Nilashi et al. [10] developed a novel MCDM method based on decision making trial and evaluation laboratory (DEMATEL) and fuzzy-TOPSIS to identify the key factors of medical tourism development in Malaysia. Literature on the selection of medical or healthy-related consumer products is scarce [11]. The relevant studies mainly focused on the selection of medical equipment and materials [11]. Recently, Gao et al. [12] developed a multi-attribute group decision making method for selecting medical consumer products in a q-rung interval-valued orthopair fuzzy environment.

From the review above, it can be seen that the MCDM method based on fuzzy set theory is widely used in the fields of medical diagnosis and health evaluation. There are few studies, however, on the selection of medical and healthcare products during epidemic outbreaks, especially the selection of antivirus masks during the COVID-19 pandemic. Hence, subsequent research should focus on the selection of antivirus masks under COVID-19 expansion to prevent individuals from acquiring them in excessive numbers and to optimise the use of protective products.

### 2.2. The MCDM Methods Based on Fuzzy Set Theories

Many scholars have put forward various MCDM methods based on the uncertainty theory with the aim of analyzing and solving decision-making problems [13,14,15,16,17,18,19,20]. The MCDM methods based on the extensions of fuzzy sets proposed by Zadeh [21] are increasingly common. In particular, the intuitionistic fuzzy set (IFS) presented by Atanassov [22] is widely applied to MCDM methods. Compared with Zadeh’s fuzzy set, which only expresses the membership degree of an element, IFS can depict both the membership (MBD) and non-membership degree (NOMBD) corresponding to the positive and negative aspects of an element. Nevertheless, there is a constraint in the field of the IFS, by which the sum of MBD and NOMBD cannot exceed 1. This constraint limits the descriptive power of INF. To overcome this constraint, Yager [23] developed a new concept of Pythagorean fuzzy set (PyFS), with a function that permits the sum of MBD and NOMBD to be greater than 1, but limits the quadratic sum of MBD and NOMBD to 1. With the extension of PyFS, more recent studies have concentrated on its basic theory [24,25] and aggregation operators [26,27,28].

The IFS and PyFS regulate the MBD and NOMBD given separately by decision makers, and the neutral membership degree (NeuMBD) depends on MBD and NOMBD. In some situations, however, where the NeuMBD needs to be given independently in practical decision-making, the IFS and PyFS fail to address the decision-making problems. In response, Cuong [29,30] proposed a new concept of picture fuzzy set (PcFS), which consists of positive membership degree (PoMBD), NeuMBD, and negative membership degree (NegMBD). PcFS tends to give diverse evaluations of the answers given by decision makers, but similar to those given by IFS. There is a restriction that the sum of PoMBD, NeuMBD, and NegMBD cannot exceed 1. Given the drawback of PcFS, Mahmood et al. [31] developed the new concept of the spherical fuzzy set (SpFS) by combining the PtFS and PcFS. SpFS is composed of PoMBD, NegMBD, and NeuMBD, where the sum of PoMBD, NegMBD, and NeuMBD is more than 1, but the quadratic sum of them is less than or equal to 1. Hence, the SpFS can handle all the uncertain information that FS, IFS, PyFS, and PcFS cannot.

### 2.3. Spherical Fuzzy Set

Based on the SpFS, Ashraf et al. [32] developed the spherical fuzzy (SpF) weighted averaging and weighted geometric aggregation operators based on Archimedean t-norm and t-conorm. Gundogdu and Kahraman [33] presented an interval-valued SpF-TOPSIS method. Jin et al. [34] defined a new concept of linguistic SpF set and introduced linguistic SpF weighted averaging and geometric operators. Gundogdu and Kahraman [35] developed a MCDM method based on Weighted Aggregated Sum Product Assessment (WASPAS) under SpF environment. Jin et al. [36] introduced the logarithmic operations into spherical fuzzy sets (SpFSs) and established some new operations and aggregation operators. Rafiq et al. [37] measured the similarity between SpF sets based on the cosine function. Zeng et al. [38] presented the notation of SpF rough set (SFRS) and built a MCDM method based on SFRS and TOPSIS. In addition, SpFS has been applied widely in practical decision-making problems. For example, Ashraf et al. [39] used the SpPFS to evaluate the influencing factors of child development, Gundogdu and Kahraman [40] addressed the problems of warehouse location selection using SpFS, and Mahmood et al. [31] discussed the application of SpFS to deal with the problems of medical diagnostics.

In reality, most natural and social laws follow normal distribution [41,42,43], such as “product life“, “climate change”, and ”stock price fluctuation“. To uncover these phenomena, Yang and Ko [41] defined the concept of normal fuzzy number (NFN), in which the inner structure consists of the mean and standard deviation of objective evaluation information. NFN not only expresses objective evaluation of target criteria, but also has higher derivative continuity and is closer to human thinking than other fuzzy numbers (FNs), such as triangular FNs, trapezoidal FNs, and hesitant FNs [42]. To fuse the objective and subjective information in MACM, Wang et al. [43] and Wang et al. [44] developed intuitionistic normal fuzzy number (INFN), which are based on two parts—IFS and NFN. The MBD of INFN signifies the degree to which an element can be described by NFN. Conversely, the NOMBD of INFN signifies the degree to which an element cannot be portrayed by NFN. Some scholars have further developed the basic theories of INFN [45,46] and presented some aggregation’s operators with their applications [47,48]. Since INFN and its relevant extensions are based on the combination of IFN and NFN, some of the drawbacks of IFN remain in INFN.

Therefore, to overcome this shortcoming, this paper proposes a novel concept of Spherical normal fuzzy set (SpNoFS), integrating the advantages of the SpFS and NFN. First, some new operation rules (score function and accuracy function) and sorting rules between SpNoFSs in a Spherical normal fuzzy environment are defined. Then, given the interrelationship between the evaluation criteria, the Bonferroni mean operator is introduced into SpNoFS to present some aggregation operators. Furthermore, a new MCDM method is developed based on the proposed aggregation operators. The selection of antivirus masks during the COVID-19 pandemic is used as an example to show the practicality and advantages of the proposed method.

## 3. Preliminaries

In this section, we briefly review the certain definitions related to fuzzy numbers and the spherical set to understand the rest of the paper clearly.

**Definition** **1** [41]. *Let R be real number collection, x,α,σ∈R, we can treat $Z=\left(\alpha ,\sigma \right)$ as a normal fuzzy number (NFN), and the membership function (MBF) of NFN can be given as *

(1) $$Z(x)=e^{-{\left(\frac{x-\alpha }{\sigma }\right)}^{2}}(\sigma >0)$$

*and the NFNs can be expressed as F.*

**Definition** **2** [48]. *Let $Z_{1}=\left(\alpha_{1},\sigma_{1}\right)$, $Z_{2}=\left(\alpha_{2},\sigma_{2}\right)$$Z_{1},Z_{2}\in F$, and λ be a nonnegative real number, we can get the operational rule of $Z_{1}$ and $Z_{2}$ as follows:*

(1) $\lambda ⊗Z_{1}=\lambda (\alpha_{1},\sigma_{1})=(\lambda \alpha_{1},\lambda \sigma_{1}),\lambda >0$, *and*

(2) $Z_{1}⊕Z_{2}=\left(\alpha_{1},\sigma_{1}\right)+\left(\alpha_{2},\sigma_{2}\right)=\left(\alpha_{1}+\alpha_{2},\sigma_{1}+\sigma_{2}\right)$.

**Definition** **3** [32]. *Let T be a finite universe collection, x∈T, then the collection *

(2) $$A=\left\{\left.\langle x,\delta_{A}(x),\gamma_{A}(x),\tau_{A}(x)\rangle \right|x\in T\right\}$$

*can be called a spherical fuzzy set (SpFS), where $\delta_{A}(x),\gamma_{A}(x),\tau_{A}(x)$ are the positive-membership degree (PoMBD), neutral-membership degree (NeuMBD), and negative membership degree (NegMBD) of x in T, respectively, and $\delta_{A}(x),\gamma_{A}(x),\tau_{A}(x)\in \left[0,1\right]$. For convenience, $A=\langle \delta_{A},\gamma_{A},\tau_{A}\rangle$ is called a spherical fuzzy number (SpFN). For any SpFN, it satisfies $0\leq \delta_{A}^{2}+\gamma_{A}^{2}+\tau_{A}^{2}\leq 1$. Then, $\pi_{A}(x)=1-\sqrt{\delta_{A}^{2}+\gamma_{A}^{2}+\tau_{A}^{2}}$ is the refusal MBF of x to T.*

**Definition** **4** [32]. *Let $A_{1}=\langle \delta_{1},\gamma_{1},\tau_{1}\rangle$ and $A_{2}=\langle \delta_{2},\gamma_{2},\tau_{2}\rangle$ be two SpFNs, and λ≥0. Then, the operation between $Z_{1}$ and $Z_{2}$ can be obtained as follows:*

(1) $A_{1}⊕A_{2}=\langle \sqrt{\delta_{1}^{2}+\delta_{2}^{2}-\delta_{1}^{2}\delta_{2}^{2}},\gamma_{1}\gamma_{2},\tau_{1}\tau_{2}\rangle$,

(2) $A_{1}⊗A_{2}=\langle \delta_{1}\delta_{2},\sqrt{\gamma_{1}^{2}+\gamma_{2}^{2}-\gamma_{1}^{2}\gamma_{2}^{2}},\sqrt{\tau_{1}^{2}+\tau_{2}^{2}-\tau_{1}^{2}\tau_{2}^{2}}\rangle$,

(3) $\lambda A_{1}=\langle \sqrt{1-{\left(1-\delta_{1}^{2}\right)}^{\lambda }},\gamma_{1}^{\lambda },\tau_{1}^{\lambda }\rangle$, *and*

(4) $A_{1}^{\lambda }=\langle \delta^{\lambda },\sqrt{1-{\left(1-\gamma_{1}^{2}\right)}^{\lambda }},\sqrt{1-{\left(1-\tau_{1}^{2}\right)}^{\lambda }}\rangle$.

## 4. The Spherical Normal Fuzzy Number and Its Operations

According to the concepts and operations of NFN and SpFN, a new concept of spherical normal fuzzy number (SpNoFN) and its operations are proposed in this section.

**Definition** **5.** *Let X be a finite set, x∈X, $A=\left(\alpha_{A},\sigma_{A}\right)\in F$, and $A=\langle \left(\alpha ,\sigma \right),\left(\delta_{A},\gamma_{A},\tau_{A}\right)\rangle$, then the collection $A=\left\{\left.\langle x,\left(\alpha_{A},\sigma_{A}\right),\left(\delta_{A}(x),\gamma_{A}(x),\tau_{A}(x)\right)\rangle \right|x\in X\right\}$ can be defined as the Spherical normal fuzzy set (SpNoFS), for which its positive-MBF is defined as*

(3) $$\delta_{A}(x)=\delta_{A}e^{-{\left(\frac{x-\alpha }{\sigma }\right)}^{2}},\;\;\;\;x\in X$$

*its neutral-MBF is defined as*

(4) $$\gamma_{A}(x)=1-(1-\gamma_{A})e^{-{\left(\frac{x-\alpha }{\sigma }\right)}^{2}},\;\;\;\;x\in X$$

*and its negative-MBF is defined as*

(5) $$\tau_{A}(x)=1-(1-\tau_{A})e^{-{\left(\frac{x-\alpha }{\sigma }\right)}^{2}},\;\;\;\;x\in X$$

*For convenience, we can call $A=\langle (\alpha_{A},\sigma_{A}),\left(\delta_{A},\gamma_{A},\tau_{A}\right)\rangle$ a spherical normal fuzzy number (SpNoFN).*

**Definition** **6.** *Let $A_{1}=\langle (\alpha_{1},\sigma_{1}),\left(\delta_{1},\gamma_{1},\tau_{1}\right)\rangle$ and $A_{2}=\langle (\alpha_{2},\sigma_{2}),\left(\delta_{2},\gamma_{2},\tau_{2}\right)\rangle$ be any two SpNoFN s, and λ≥0, we define*

(1) $A_{1}⊕A_{2}=\left(\left(\alpha_{1}+\alpha_{2},\sigma_{1}+\sigma_{2}\right),\sqrt{\delta_{1}^{2}+\delta_{2}^{2}-\delta_{1}^{2}\delta_{2}^{2}},\gamma_{1}\gamma_{2},\tau_{1}\tau_{2}\right)$,

(2) $A_{1}⊗A_{2}=\left(\begin{array}{c} \left(\alpha_{1}\alpha_{2},\alpha_{1}\alpha_{2}\sqrt{\frac{\sigma_{1}^{2}}{\alpha_{1}^{2}}+\frac{\sigma_{2}^{2}}{\alpha_{2}^{2}}}\right),\\ \delta_{1}\delta_{2},\sqrt{\gamma_{1}^{2}+\gamma_{2}^{2}-\gamma_{1}^{2}\gamma_{2}^{2}},\sqrt{\tau_{1}^{2}+\tau_{2}^{2}-\tau_{1}^{2}\tau_{2}^{2}} \end{array}\right)$,

(3) $\lambda A_{1}=\left(\left(\lambda \alpha_{1},\lambda \sigma_{1}\right),\left(\sqrt{1-{\left(1-\delta_{1}^{2}\right)}^{\lambda }},\gamma_{1}^{\lambda },\tau_{1}^{\lambda }\right)\right)$, *and*

(4) $A_{1}^{\lambda }=\left(\left(\alpha_{1}^{\lambda },\lambda^{\frac{1}{2}}\alpha_{1}^{\lambda -1}\sigma_{1}\right),\delta_{1}^{\lambda },\sqrt{1-{\left(1-\gamma_{1}^{2}\right)}^{\lambda }},\sqrt{1-{\left(1-\tau_{1}^{2}\right)}^{\lambda }}\right)$.

**Proposition** **1.** *Let $A_{1}=\langle (\alpha_{1},\sigma_{1}),\left(\delta_{1},\gamma_{1},\tau_{1}\right)\rangle$, $A_{2}=\langle (\alpha_{2},\sigma_{2}),\left(\delta_{2},\gamma_{2},\tau_{2}\right)\rangle$, $A_{3}=\langle (\alpha_{3},\sigma_{3}),\left(\delta_{3},\gamma_{3},\tau_{3}\right)\rangle$ be any three SpNoFNs, and $\lambda ,\lambda_{1},\lambda_{2}\geq 0$, there are some characters that can be described as follows:*

(1) $A_{1}⊕A_{2}=A_{2}⊕A_{1}$,

(2) $\left(A_{1}⊕A_{2}\right)⊕A_{3}=A_{1}⊕\left(A_{2}⊕A_{3}\right)$,

(3) $A_{1}⊗A_{2}=A_{2}⊗A_{1}$,

(4) $\left(A_{1}⊗A_{2}\right)⊗A_{3}=A_{1}⊗\left(A_{2}⊗A_{3}\right)$,

(5) $\lambda_{1}A_{1}⊕\lambda_{2}A_{1}=\left(\lambda_{1}⊕\lambda_{2}\right)A_{1}$,

(6) $\lambda \left(A_{1}⊕A_{2}\right)=\lambda A_{1}⊕\lambda A_{2}$,

(7) ${\left(A_{1}^{\lambda_{1}}\right)}^{\lambda_{2}}=A_{1}^{\lambda_{1}\lambda_{2}}$*, and*

(8) $A_{1}^{\lambda_{1}}⊗A_{1}^{\lambda_{2}}=A_{1}^{\lambda_{1}+\lambda_{2}}$.

**Proof.**  See Appendix A. □

**Definition** **7.** *Let $A=\langle (\alpha ,\sigma ),\left(\delta ,\gamma ,\tau \right)\rangle$ be a SpNoFN, its score function is described as $S_{1}(A)=1+\alpha \left(\delta^{2}-\gamma^{2}-\tau^{2}\right)$ and $S_{2}(A)=1+\sigma \left(\delta^{2}-\gamma^{2}-\tau^{2}\right)$, while its accuracy function is described as $H_{1}(A)=\alpha \left(\delta^{2}+\gamma^{2}+\tau^{2}\right)$ and $H_{2}(A)=\sigma \left(\delta^{2}+\gamma^{2}+\tau^{2}\right)$.*

Based on these scores and accuracy functions, we define the order relation between the given SpNoFNs to rank them, as below.

**Definition** **8.** *For two SpNoFNs, $A_{1}=\langle (\alpha_{1},\sigma_{1}),\left(\delta_{1},\gamma_{1},\tau_{1}\right)\rangle$ and $A_{2}=\langle (\alpha_{2},\sigma_{2}),\left(\delta_{2},\gamma_{2},\tau_{2}\right)\rangle$ and their score and accuracy functions are computed by using Definition 7, then an order relation, denoted by >, between the given SpNoFNs is defined as*

(1) *If $S_{1}(A_{1})>S_{1}(A_{2})$, then $A_{1}>A_{2}$,*

(2) *If $S_{1}(A_{1})=S_{1}(A_{2})$ and $H_{1}(A_{1})>H_{1}(A_{2})$, then $A_{1}>A_{2}$,*

(3) *If $S_{1}(A_{1})=S_{1}(A_{2})$ and $H_{1}(A_{1})=H_{1}(A_{2})$, then*

*If $S_{2}(A_{1})<S_{2}(A_{2})$, then $A_{1}>A_{2}$,*

*If $S_{2}(A_{1})=S_{2}(A_{2})$ and $H_{2}(A_{1})<H_{2}(A_{2})$, then $A_{1}>A_{2}$.*

**Definition** **9.** [49] *Let p≥0,q≥0, and p+q>0, $a_{i}\;\;(i=1,2,\cdots ,n)$ be a set of non-negative real numbers, then the function*

(6) $$BM(A_{1},A_{2},\cdots ,A_{n})={\left(\frac{1}{n(n-1)}\sum_{\begin{array}{c} i,j=1\\ i\neq j \end{array}}^{n}a_{i}^{p}a_{j}^{q}\right)}^{\frac{1}{p+q}}$$

*is called the Bonferroni mean operator.*

## 5. Spherical Normal Fuzzy Bonferroni Mean Operators

In this section, the Bonferroni mean can be introduced into the SpNoF environment, and according to the operations of SpNoFN and the SpNoF, Bonferroni mean operators are proposed as follows:

**Definition** **10.** *Let $A_{i}=\langle (\alpha_{i},\sigma_{i}),\left(\delta_{i},\gamma_{i},\tau_{i}\right)\rangle \;\;(i=1,2,\cdots ,n)$ be a set of SpNoFNs. Then, the Spherical normal fuzzy Bonferroni mean (SpNoFBM) operator is defined as *

(7) $$SpNoFBM\left(A_{1},A_{2},\cdots ,A_{n}\right)={\left(\frac{1}{n(n-1)}\sum_{i=1}^{n}\sum_{j=1}^{n}{\left(A_{i}\right)}^{p}⊗{\left(A_{j}\right)}^{q}\right)}^{\frac{1}{p+q}}$$

**Theorem** **1.** *Let $A_{i}=\langle (\alpha_{i},\sigma_{i}),\left(\delta_{i},\gamma_{i},\tau_{i}\right)\rangle \;\;(i=1,2,\cdots ,n)$ be a set of SpNoFNs, then the value calculated by SpNoFBM operator is also a SpNoFN, that is*

(8) $$\begin{array}{c} \;SpNoFBM\left(A_{1},A_{2},\cdots ,A_{n}\right)\\ =\left[\begin{array}{l} \left(\frac{1}{n(n-1)}\sum_{\begin{array}{c} i,j=1\\ i\neq j \end{array}}^{n}\alpha_{i}^{p}\alpha_{j}^{q},\sqrt{\frac{1}{p+q}}{\left(\frac{1}{n(n-1)}\sum_{\begin{array}{c} i,j=1\\ i\neq j \end{array}}^{n}\alpha_{i}^{p}\alpha_{j}^{q}\right)}^{\frac{1}{p+q}-1}\left(\frac{1}{n(n-1)}\sum_{\begin{array}{c} i,j=1\\ i\neq j \end{array}}^{n}\alpha_{i}^{p}\alpha_{j}^{q}\sqrt{\frac{p\sigma_{i}^{2}}{\alpha_{i}^{2}}+\frac{q\sigma_{j}^{2}}{\alpha_{j}^{2}}}\right)\right),\\ \left(\begin{array}{l} {\left(\sqrt{1-{\left(\prod_{\begin{array}{c} i,j=1\\ i\neq j \end{array}}^{n}\left(1-{\left(\delta_{i}^{2}\right)}^{p}{\left(\delta_{j}^{2}\right)}^{q}\right)\right)}^{\frac{1}{n(n-1)}}}\right)}^{\frac{1}{p+q}},\sqrt{1-{\left(1-{\left(\prod_{\begin{array}{c} i,j=1\\ i\neq j \end{array}}^{n}\left(1-{\left(1-\gamma_{i}^{2}\right)}^{p}{\left(1-\gamma_{j}^{2}\right)}^{q}\right)\right)}^{\frac{1}{n(n+1)}}\right)}^{\frac{1}{p+q}}},\\ \sqrt{1-{\left(1-{\left(\prod_{\begin{array}{c} i,j=1\\ i\neq j \end{array}}^{n}\left(1-{\left(1-\tau_{i}^{2}\right)}^{p}{\left(1-\tau_{j}^{2}\right)}^{q}\right)\right)}^{\frac{1}{n(n-1)}}\right)}^{\frac{1}{p+q}}} \end{array}\right] \end{array}\right. \end{array}$$

**Proof.**  See Appendix B. □

We find that the SpNoFBM operator has some properties as follows:

**Theorem** **2.** *(Idempotency). Supposing for $A_{i}=\langle (\alpha_{i},\sigma_{i}),\left(\delta_{i},\gamma_{i},\tau_{i}\right)\rangle \;\;(i=1,2,\cdots ,n)$ as equal with A, then $SpNoFBM\left(A_{1},A_{2},\cdots ,A_{n}\right)=A$.*

**Proof.**  See Appendix C. □

**Theorem** **3.** *(Boundedness). Let $A_{i}=\langle (\alpha_{i},\sigma_{i}),\left(\delta_{i},\gamma_{i},\tau_{i}\right)\rangle \;\;(i=1,2,\cdots ,n)$ be a set of SpNoFNS. Supposing $A^{-}=\underset{\leq i\leq n}{\min}\left\{A_{i}\right\},A^{+}=\underset{\leq i\leq n}{\max}\left\{A_{i}\right\}$, then $A^{-}\leq SpNoFBM\left(A_{1},A_{2},\cdots ,A_{n}\right)\leq A^{+}$.*

**Proof.**  See Appendix C. □

**Theorem** **4.** *(Monotonicity). Suppose $\left(A_{1},A_{2},\cdots ,A_{n}\right)$ and $\left(B_{1},B_{2},\cdots ,B_{n}\right)$ are two collections of SpNoFNs, $A_{i}=\langle (\alpha_{A_{i}},\sigma_{A_{i}}),\left(\delta_{A_{i}},\gamma_{A_{i}},\tau_{A_{i}}\right)\rangle \;\;(i=1,2,\cdots ,n)$, and $B_{i}=\langle (\alpha_{B_{i}},\sigma_{B_{i}}),\left(\delta_{B_{i}},\gamma_{B_{i}},\tau_{B_{i}}\right)\rangle \;\;(i=1,2,\cdots ,n)$, (i=1,2,⋯,n). For ∀i, if $\alpha_{A_{i}}\leq \alpha_{B_{i}}$ and $\delta_{A_{i}}\leq \delta_{B_{i}},\gamma_{A_{i}}\geq \gamma_{B_{i}},\tau_{A_{i}}\geq \tau_{B_{i}}$, then $SpNoFBM\left(A_{1},A_{2},\cdots ,A_{n}\right)\leq SpNoFBM\left(B_{1},B_{2},\cdots ,B_{n}\right)$.*

**Proof.**  See Appendix C. □

**Definition** **11.** *Let $A_{i}=\langle (\alpha_{i},\sigma_{i}),\left(\delta_{i},\gamma_{i},\tau_{i}\right)\rangle \;\;(i=1,2,\cdots ,n)$ be a set of SPNOFNs, $W=\left(w_{1},w_{2},\cdots ,w_{n}\right)$ be the weight vector of $A_{i}$, where $w_{i}\geq 0$, and $\sum_{i=1}^{n}w_{i}=1$. The Spherical normal fuzzy weighed Bonferroni mean (SpNoFWBM) operator is defined as *

(9) $$SpNoFWBM\left(A_{1},A_{2},\cdots ,A_{n}\right)={\left(\frac{1}{n(n-1)}\sum_{i=1}^{n}\sum_{j=1}^{n}{\left(A_{i}w_{i}\right)}^{p}⊗{\left(A_{j}w_{j}\right)}^{q}\right)}^{\frac{1}{p+q}}.$$

**Theorem** **5.** *Let $A_{i}=\langle (\alpha_{i},\sigma_{i}),\left(u_{i},\eta_{i},v_{i}\right)\rangle \;\;(i=1,2,\cdots ,n)$ be a set of SpNoFNs, then the aggregated value obtained by the SpNoFWBM operator is also a SpNoFN and is given as*

(10) $$\begin{array}{l} SpNoFWBM\left(A_{1},A_{2},\cdots ,A_{n}\right)\\ =\left[\begin{array}{l} \left(\begin{array}{l} {\left(\frac{1}{n(n-1)}\sum_{\begin{array}{c} i,j=1\\ i\neq j \end{array}}^{n}{\left(\alpha_{i}w_{i}\right)}^{p}{\left(\alpha_{j}w_{j}\right)}^{q}\right)}^{\frac{1}{p+q}},\sqrt{\frac{1}{p+q}}{\left(\frac{1}{n(n-1)}\sum_{\begin{array}{c} i,j=1\\ i\neq j \end{array}}^{n}{\left(\alpha_{i}w_{i}\right)}^{p}{\left(\alpha_{j}w_{j}\right)}^{q}\right)}^{\frac{1}{p+q}-1}\cdot \\ \frac{1}{n(n-1)}\sum_{\begin{array}{c} i,j=1\\ i\neq j \end{array}}^{n}{\left(\alpha_{i}w_{i}\right)}^{p}{\left(\alpha_{j}w_{j}\right)}^{q}\sqrt{\frac{p{\left(\sigma_{i}w_{i}\right)}^{2}}{{\left(\alpha_{i}w_{i}\right)}^{2}}+\frac{q{\left(\sigma_{j}w_{j}\right)}^{2}}{{\left(\alpha_{j}w_{j}\right)}^{2}}} \end{array}\right),\\ \left(\begin{array}{l} {\left(\sqrt{1-{\left(\prod_{\begin{array}{c} i,j=1\\ i\neq j \end{array}}^{n}\left(1-{\left(1-{\left(1-\delta_{i}^{2}\right)}^{w_{i}}\right)}^{p}{\left(1-{\left(1-\delta_{j}^{2}\right)}^{w_{j}}\right)}^{q}\right)\right)}^{\frac{1}{n(n-1)}}}\right)}^{\frac{1}{p+q}},\sqrt{1-{\left(1-{\left(\prod_{\begin{array}{c} i,j=1\\ i\neq j \end{array}}^{n}\left(1-{\left(1-{\left(\gamma_{i}^{w_{i}}\right)}^{2}\right)}^{p}\right)\left(1-{\left(1-{\left(\gamma_{j}^{w_{j}}\right)}^{2}\right)}^{q}\right)\right)}^{\frac{1}{n(n-1)}}\right)}^{\frac{1}{p+q}}},\\ \sqrt{1-{\left(1-{\left(\prod_{\begin{array}{c} i,j=1\\ i\neq j \end{array}}^{n}\left(1-{\left(1-{\left(\tau_{i}^{w_{i}}\right)}^{2}\right)}^{p}\right)\left(1-{\left(1-{\left(\tau_{j}^{w_{j}}\right)}^{2}\right)}^{q}\right)\right)}^{\frac{1}{n(n-1)}}\right)}^{\frac{1}{p+q}}} \end{array}\right) \end{array}\right. \end{array}$$

**Proof.**  See Appendix D. □

Similar to the SpNoFBM operator, the proposed SpNoFWBM operator also satisfies the properties such as idempotency, monotonicity, and boundedness.

## 6. A Novel MCDM Model Based on Proposed Aggregation Operators

**Figure 1:**
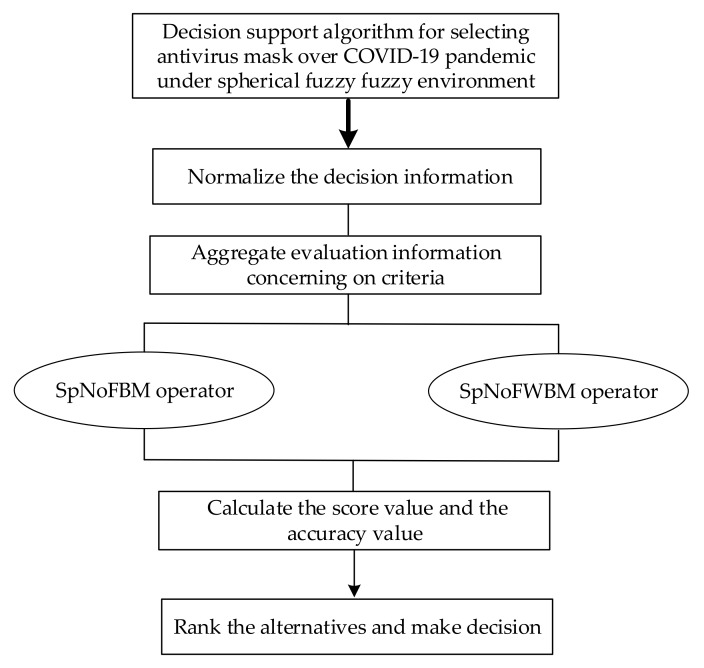
The procedure of the proposed method.

In this section, a novel MCDM model based on SpNoFBM operator and SpNoFWBM operator are proposed under the SpNoF information environment. The $A=\left\{A_{1},A_{2},\cdots ,A_{n}\right\}$ represents a collection of n alternatives, the $C=\left\{C_{1},C_{2},\cdots ,C_{m}\right\}$ represents a collection of m criteria which are used to evaluate the alternatives, and the corresponding weight of criterion is $w=\left\{w_{1},w_{2},\cdots ,w_{m}\right\}$, and $w_{i}\geq 0$, and $\sum_{i=1}^{n}w_{i}=1$. We suppose the evaluation information of criterion $C_{j}$ concerning the alternative $A_{i}$ as SpNoFN $A_{ij}=\langle (\alpha_{ij},\sigma_{ij}),\left(\delta_{ij},\gamma_{ij},\tau_{ij}\right)\rangle$ (i=1,2,⋯,n;  j=1,2,⋯,m). Then, $\delta_{ij}$ stands for the PoMBD to which alternative $A_{i}$ can be described by NFN $\left(\alpha_{ij},\sigma_{ij}\right)$ with respect to $C_{j}$, $\gamma_{ij}$ and $\tau_{ij}$ denote the NeuMBD, NegMBD to which alternative $A_{i}$ can be described by NFN $\left(\alpha_{ij},\sigma_{ij}\right)$ with respect to $C_{j}$, respectively. In addition, the decision information matrix $A={\left(A_{ij}\right)}_{n\times m}$, which consists of a set of n alternatives and a set of m criteria, is constructed. The new MCDM model based on a SpNoFBM operator and SpNoFWBM operator is proposed by considering the interrelationships between criteria, as shown in Figure 1, and the procedures of the proposed model are summarized as follows:

**Step 1.** Normalize the decision information

To eliminate the influence of different types of criteria on decision results, the original decision matrix $A={\left(A_{ij}\right)}_{n\times m}$ should be normalized to $\bar{A}={\left({\bar{A}}_{ij}\right)}_{n\times m}$ and obtain the same type of criteria.

For benefit type of criteria [45]:

(11) $${\bar{\alpha }}_{ij}=\frac{\alpha_{ij}}{\underset{i}{\max}(\alpha_{ij})},\;\;{\bar{\sigma }}_{ij}=\frac{\sigma_{ij}}{\underset{i}{\max}(\sigma_{ij})}\cdot \frac{\sigma_{ij}}{\alpha_{ij}},\;\;\;\;{\bar{\delta }}_{ij}=\delta_{ij},{\bar{\gamma }}_{ij}=\gamma_{ij},{\bar{\tau }}_{ij}=\tau_{ij}.$$

For cost type of criteria [36]:

(12) $${\bar{\alpha }}_{ij}=\frac{\underset{i}{\min}(\alpha_{ij})}{\alpha_{ij}},\;\;{\bar{\sigma }}_{ij}=\frac{\sigma_{ij}}{\underset{i}{\max}(\alpha_{ij})}\cdot \frac{\sigma_{ij}}{\alpha_{ij}},\;\;\;\;{\bar{\delta }}_{ij}=\delta_{ij},{\bar{\gamma }}_{ij}=\gamma_{ij},{\bar{\tau }}_{ij}=\tau_{ij}.$$

**Step 2.** Aggregate evaluation information concerning on criteria

The evaluation formation of ${\bar{A}}_{ij}=\langle \left({\bar{\alpha }}_{ij},{\bar{\sigma }}_{ij}),({\bar{\delta }}_{ij},{\bar{\gamma }}_{ij},{\bar{\tau }}_{ij}\right)\rangle \;\;$ can be aggregated into the synthetic value ${\bar{A}}_{i}=\langle \left({\bar{\alpha }}_{i},{\bar{\sigma }}_{i}),({\bar{\delta }}_{i},{\bar{\gamma }}_{i},{\bar{\tau }}_{i}\right)\rangle \;\;$ concerning on alternative $A_{i}$ by the SpNoFBM operator, or the synthetic value ${\bar{A}}_{i}=\langle \left({\bar{\alpha }}_{i},{\bar{\sigma }}_{i}),({\bar{\delta }}_{i},{\bar{\gamma }}_{i},{\bar{\tau }}_{i}\right)\rangle \;\;$ with decision makers’ preference by the SpNoFWBM operator.

**Step 3.** Calculate the score value $S\left({\bar{A}}_{i}\right)$ and the accuracy value $H\left({\bar{A}}_{i}\right)$ of $A_{i}$ according to Definition 7.

**Step 4.** Rank the alternatives based on Definition 8, the score value $S\left({\bar{A}}_{i}\right)$, and the accuracy value $H\left({\bar{A}}_{i}\right)$.

**Step 5.** Make the decision for selecting the optimal alternative based on the ranking result.

## 7. The Case on Antivirus Mask Selecting over the COVID-19 Pandemic

In this section, to verify the practicability of the proposed model, we consider issues of selecting an antivirus mask, of which the description can be read as follows.

### 7.1. Decision Procedure

In the current severe case of COVID-19 transmission [3,4], there are six types of masks that are commonly available in the market, including medical surgical masks, particulate respirators (N95/KN95 and above), medical protective masks, disposable medical masks, ordinary non-medical masks, and gas masks. One person needs to buy an antivirus mask from the above six candidate antivirus masks. In addition, he or she evaluates the antivirus masks by considering four criteria, namely, leakage rate ($C_{1}$), that is the adhesiveness of the mask structure design to cover the human face; reusability ($C_{2}$); quality of raw materials ($C_{3}$); and filtration efficiency ($C_{4}$), which means the filtration efficiency of non-oily 0.3μm particles is greater than 95%, and it must also have medical protection requirements such as surface moisture resistance and blood barrier. Due to the influence of peoples’ preference on the evaluation result, the weight $w_{i}$ of criterion provided by the decision maker showed as w=(0.25,0.2,0.3,0.25). The people give the evaluation value of criterion $C_{i}\;\;\left(i=1,2,3,4\right)$ concerning each $S_{i}\;\;\left(i=1,2,3,4,5,6\right)$ based on the SpNoFS, and the evaluation information can be summarized to form a decision information matrix, as shown in Table 1. Then, the main aim of the decision maker is to select the best antivirus mask based on the decision information matrix.

**Step 1:** Since all the criteria are benefit type of criteria, according to formula (10), we transform the original decision information matrix in Table 1 into the normalized decision information matrix shown in Table 2.

**Step 2:** Apply the proposed SpNoFWBM operator in definition 11 to aggregate evaluation information concerning the criterions in Table 2 (p=q=2). Then, we obtain the overall evaluation values as follows:

${\bar{A}}_{1}=$<(0.179,0.015), 0.168, 0.636, 0.743>, ${\bar{A}}_{2}=$<(0.189, 0.019), (0.306, 0.675, 0.674)>,

${\bar{A}}_{3}=$<(0.168, 0.015), (0.306, 0.674, 0.681)>, ${\bar{A}}_{4}=$<(0.184, 0.017), (0.312, 0.649, 0.605)>,

${\bar{A}}_{5}=$<(0.92, 0.016), (0.177, 0.738, 0.65)>, ${\bar{A}}_{6}=$<(0.181, 0.016), (0.21, 0.713, 0.598)>.

**Step 3:** According to definition 7, we can calculate the score value of each of the alternatives as follows:

$S(A_{1})$ = 0.834, $S(A_{2})$ = 0.845, $S(A_{3})$ = 0.861,$S(A_{4})$ = 0.873, $S(A_{5})$ = 0.82, $S(A_{6})$ = 0.852.

**Step 4:** By definition 8 and score value, we can get the alternative ranking as $A_{4}>A_{3}>A_{6}>A_{2}>A_{1}>A_{5}$, that is disposable medical mask > medical protective mask > gas mask > particulate respirator > medical surgical mask > ordinary non-medical mask.

**Step 5:** Based on the obtained ordering, we can conclude that a disposable medical mask is the best choice; while the medical protective mask is the second priority among the given alternatives.

### 7.2. Sensitive Analysis

In this part, we examine the impacts of parameters p,q and w in SpNoFWBM operator on the decision result.

**Figure 2:**
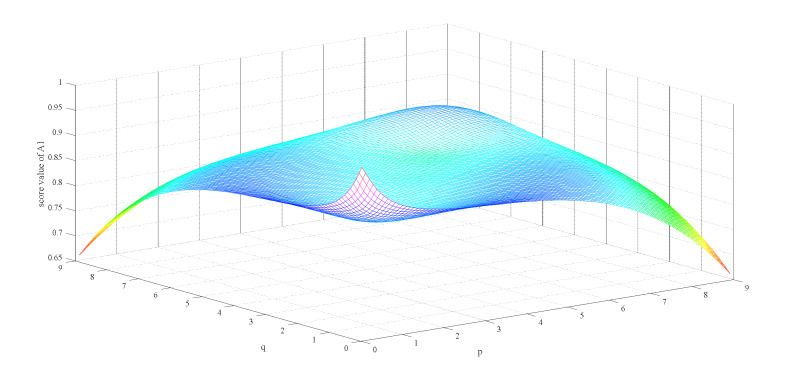
Score value of medical surgical mask ($A_{1}$) when $p,\;\;\;q\in \left[0.1,9\right]$.

**Figure 3:**
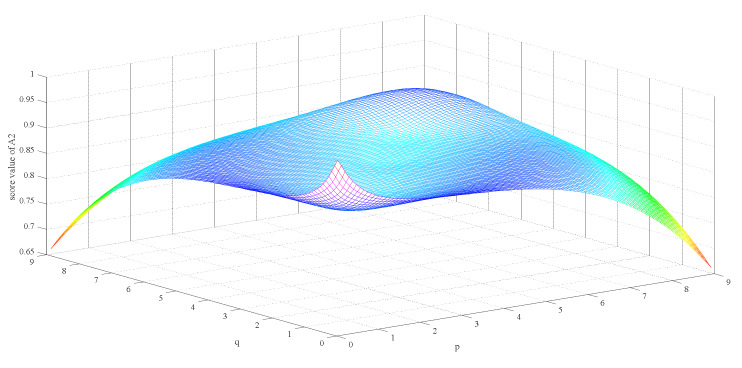
Score value of particulate respirator ($A_{2}$) when $p,\;\;\;q\in \left[0.1,9\right]$.

**Figure 4:**
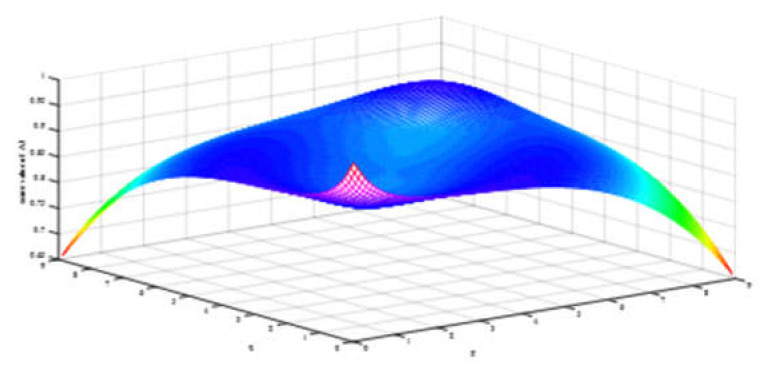
Score value of medical protective mask ($A_{3}$) when $p,\;\;\;q\in \left[0.1,9\right]$.

**Figure 5:**
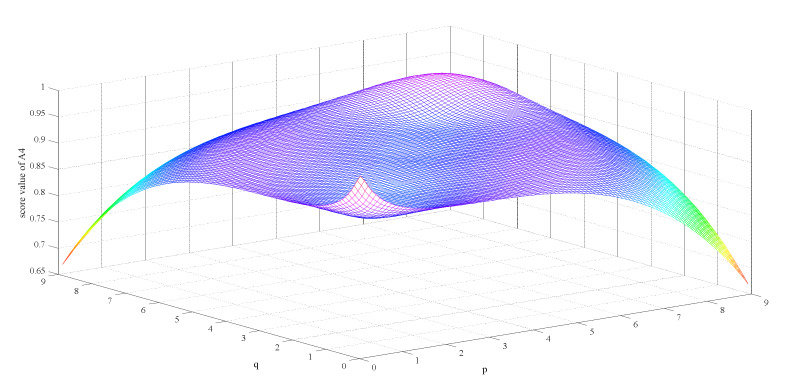
Score value of disposable medical mask ($A_{4}$) when $p,\;\;\;q\in \left[0.1,9\right]$.

**Figure 6:**
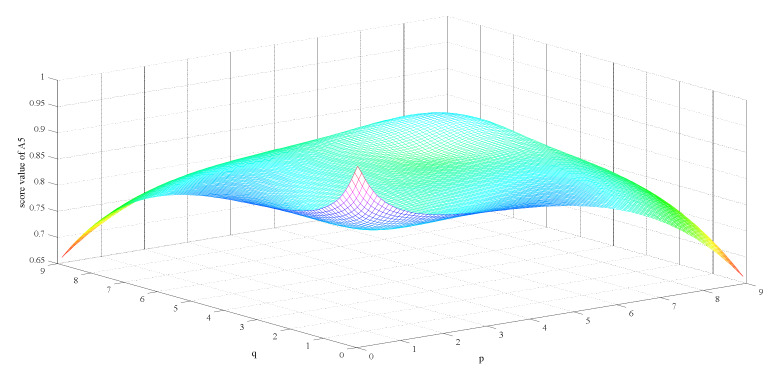
Score value of ordinary non-medical mask ($A_{5}$) when $p,\;\;\;q\in \left[0.1,9\right]$.

**Figure 7:**
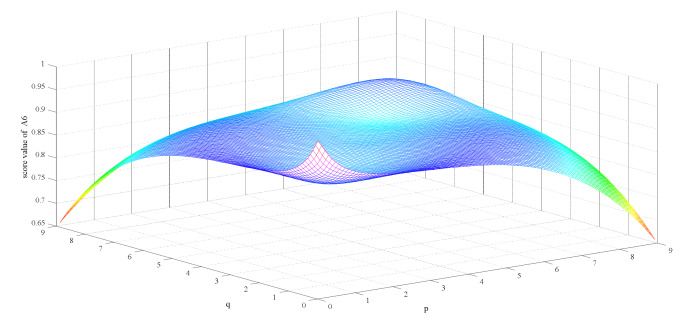
Score value of gas mask ($A_{6}$) when $p,\;\;\;q\in \left[0.1,9\right]$.

Firstly, we observe the impact of parameters p,q on the ranking result. Table 3 shows the impact of the simultaneous changes of parameters p,q on the alternative ranking. Figure 2, Figure 3, Figure 4, Figure 5, Figure 6 and Figure 7 show the effect of the synchronous changes of parameters p,q on the score value of each alternative.

According to Table 3, when p is equal to q, such as p,q = 0.3, 1, or 9, the ranking is $A_{4}>A_{3}>A_{6}>A_{2}>A_{1}>A_{5}$; when p,q = 1, the ranking is $A_{4}>A_{3}>A_{2}>A_{6}>A_{1}>A_{5}$, the best choice is always $A_{4}$, and the worst best is $A_{5}$. This indicates that there are no significant changes in the field of alternative ranking with synchronous change of parameters p,q(p=q). When p is not equal to q, for example, p = 0.1, q = 5, the alternative ranking is $A_{3}>A_{4}>A_{2}>A_{6}>A_{1}>A_{5}$ and the best choice is $A_{3}$. However, when p = 5, q = 0.1, the ranking results are significantly changed into $A_{4}>A_{3}>A_{2}>A_{6}>A_{1}>A_{5}$; the best choice accordingly changes into $A_{4}$, which means that the alternative ranking changes along with the simultaneous variation of parameters p,q(p≠q).

Based on Figure 2, Figure 3, Figure 4, Figure 5, Figure 6 and Figure 7, we also find that there are noteworthy correlations between the score values of the six alternatives and the synchronous change of parameters p,q. For example, with the changes of p,q from small to large, the score values of the six alternatives vary inversely from large to small.

**Figure 8:**
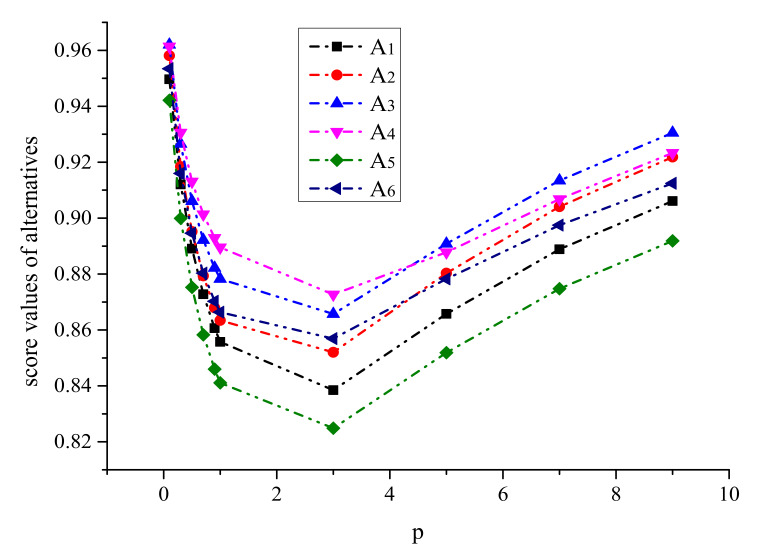
Alternatives ranking with q=2) when $p,\;\;\;q\in \left[0.1,9\right]$.

**Figure 9:**
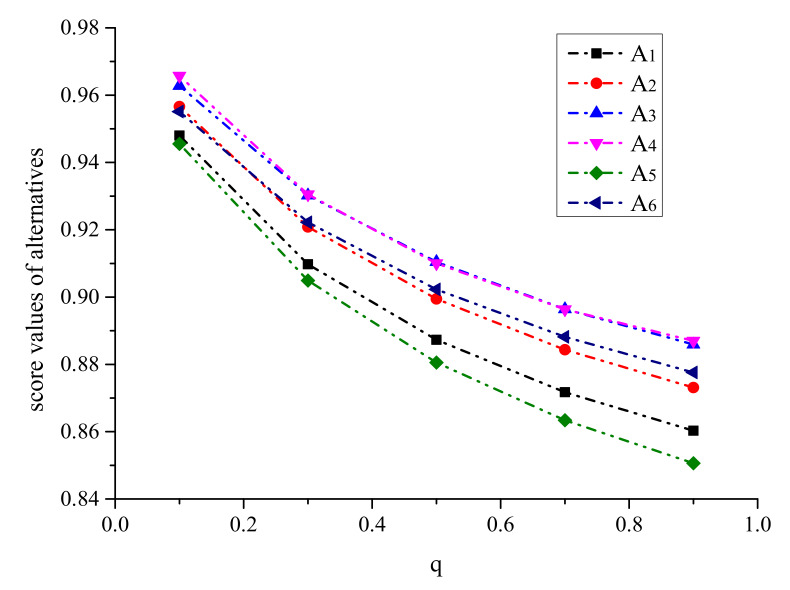
Alternatives ranking with p=2 and $q\in \left[0.1,0.9\right]$.

In addition, as show in Figure 8 and Figure 9, when one of the values of p and q is fixed, while another is changed, then we discover that the alternative ranking has altered with the variation of p or q. Hence, it is concluded that the parameters p,q in the SpNoFWBM operator greatly affect the decision results. As such, people can adjust the values of p and q based on their subjective preferences to get a different alternative ranking and make a rational consumption decision.

The criterion weight denotes that the different groups’ preference directly affects the peoples’ decision-making. Hence, the impact of criterion weight $w_{i}\;\;(i=1,2,3,4)$ on alternatives ranking is discussed. The results are shown in Table 4. For ordinary people, we should attach more importance to the leakage rate ($C_{1}$) and reusability ($C_{2}$). As such, we can set $w_{1}$ = 0.85, $w_{2}$ = $w_{3}$ = $w_{4}$ = 0.05 or $w_{2}$ = 0.85, $w_{1}$ = $w_{3}$ = $w_{4}$ = 0.05, and get the disposable medical mask as the optimal decision. For medical workers, we should be more concerned about the quality of the raw materials ($C_{3}$) and filtration efficiency ($C_{4}$), i.e., when $w_{3}$ = 0.85, $w_{1}$ = $w_{2}$ = $w_{4}$ = 0.05, the corresponding ranking is $A_{3}>A_{2}>A_{6}>A_{5}>A_{4}>A_{1}$ and a medical protective mask is the best choice; and, when $w_{4}$ = 0.85, $w_{1}$ = $w_{2}$ = $w_{3}$ = 0.05, a medical surgical mask is the final decision. These results show that a different set of criterion weights have a remarkable impact on the ranking of the six candidates. With these concerns, people can make reasonable product purchase decisions with actual utility maximization using the proposed method.

### 7.3. Comparative Analysis

In order to verify the superiorities of the proposed algorithm, in this sub-section, we compared the proposed algorithm with some methods based on SpFS, PtFS, PyFS, and INFS, respectively. The characteristic comparisons of the different methods are shown in Table 5.

Compared with existing methods based on PtFS, INFS. As shown in Table 4, the methods proposed by Wang et al. [44] and Yang et al. [47] combined the IFS with NFN, and the method by Zhang et al. [48] considered the correlations of different variables under INFS, but those methods only rely on IFS. As mentioned above, the IFS only use two indices (MBD and NOMBD) to portray the structure of evaluation information that cannot obtain any rational decision result in some practical decision problems. The same shortcomings exist in Yager [23]’s method based on PyFS. Although, Cuong [29,30]’s method introduced a new membership, named NeuMBD, to overcome the shortage of the INF. Tt also has its constraint condition that is the sum of PoMBD, NegMBD and NeuMBD cannot exceed 1, which cannot address more complex information. In order to overcome such shortcomings and considering normal distribution characteristics of objective information in real life, our proposed method combined the IFS, PyFS, PtFS, SpFN, and NFN. It is able to handle more complex decision information and has broader applications than abovementioned methods.

Further, to compare the difference between ranking results derived from existing methods and our method, we use the Spearman’s rank-correlation test [50] to conduct the following discussions. In it, two key test statistics $r_{s}$ and *Z* are defined as

(13) $$r_{s}=1-6\sum_{i=1}^{n}{\left(d_{i}\right)}^{2}/n\left(n^{2}-1\right)$$

(14) $$Z=r_{s}\sqrt{n-1},$$

where *n* denotes the number of alternatives and $d_{i}$ signifies the difference degree of each alternative between two different rankings. $r_{s}\in \left[-1,1\right]$, the closer this value of $\left|r_{s}\right|$ is to 1, the stronger the relationship between the two rankings. What is more, a positive relationship exists between two rankings if *Z* ≥ 1.645, or else they are dissimilar [46].

The Spearman’s rank-correlations test results are shown in Table 6. According to Table 6, we can see the similarities and differences between the methods by [32,36,37,45], and our method.

Specifically, the method based on Archimedean operators proposed by Ashraf et al. [23] is applied in this numerical example. The result of an alternative ranking is $A_{4}>A_{6}>A_{3}>A_{2}>A_{1}>A_{5}$. The method using logarithmic operations presented by Jin et al. [36] can be used to analyze this example. The ranking is $A_{4}>A_{3}>A_{2}>A_{6}>A_{1}>A_{5}$. The ranking results based on the method by Rafiq et al. [37] is $A_{4}>A_{2}>A_{1}>A_{3}>A_{5}>A_{6}$. These results show that the best choice is consistent with the method proposed here, but not the overall ranking. Moreover, the ranking result based on the method in [45] is $A_{3}>A_{1}>A_{4}>A_{2}>A_{5}>A_{6}$, which is different from the results of the method proposed here, both in overall ranking and in best choice. Furthermore, from the Spearman’s rank-correlation test, it can be inferred that the rankings produced by the methods in [32,36] are similar to that produced by the proposed method, and the ranking using methods [37,45] are uncorrelated with the ranking based on the proposed method.

The same best choice indicates that the proposed method is effective. The different overall ranking manifests the otherness between the proposed method and the existing methods. The causes of the difference can be summarized as follows. One of the causes is the expression of evaluation information. The methods developed by [32,36,37] can only describe the subjective evaluation information given by decision makers (SpFN); they neglect the objective information of the target criterion (NFN). In contrast, the proposed method, which combines SpFN and NFN, can depict both the subjective and the objective evaluation information. Another cause is the process of information aggregation. The method in [32,36,37,45] assumes that the criteria of alternatives are independent, which is inconsistent with some real situations. In contrast, the proposed method examined the interrelationship between different criteria. It has greater descriptive capability for decision information and a reasonable decision process.

## 8. Conclusions

To sum up, for the purpose of remedying the weakness of SpFN and NFN, our study develops a new concept of the SpNoFN which does not only express the multiple types of evaluation answers of PoMBD, NeuMBD, and NegMBD from decision makers, but also considers the objective information of the assessment objects. Moreover, we defined the new score function and accuracy function, as well as its operations. Due to the complement of the BM operator, we introduce it in connection with the SpNoFN environment and the proposed SpNoFBM operator and SpNoFWBM operator to investigate the interrelationship between input arguments and criteria. Based on the proposed SpNoFBM operator or SpNoFWBM operator, we also construct a MCDM method that is applied to the actual case on antivirus mask selection over the COVID-19 pandemic to illustrate its efficiency. Additionally, the comparative analysis based on the case is given to verify its advantages over existing models. In general, the proposed method has the following implications:

(1) The proposed method simultaneously considers both subjective evaluation information of decision makers and objective information of target criteria by combining SpFN with NFN. Compared with the existing methods, our methods are more general and powerful.
(2) The proposed MCMD method and information aggregation operators are based on the BM operator; it pays more attention to the interrelationship between any two different SpNoFNs and also to the influence of the interrelationships on the decision result. The decision procedure of our proposed method is more in line with the real situation.
(3) There are three parameters, namely p,q, and w, in the proposed method, the value of which can be adjusted by the decision makers based on subjective preferences and real situation to obtain corresponding decision results. As such, the method of our study renders the process of decision and information aggregation more flexible.

In future studies, more information aggregation operators under SpNoFN environment, such as spherical normal fuzzy Hamy mean operators and spherical normal fuzzy interaction operators, should be investigated. The application of the proposed method can also be extended into medical diagnosis, disease recognition, brain hemorrhage, and other healthcare problems.

## Figures and Tables

**Table 1:** Original decision information matrix

	$C_{1}$	$C_{2}$	$C_{3}$	$C_{4}$
medical surgical mask ($A_{1}$) 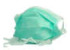	<(135,11.8), (0.29,0.54,0.61)>	<(48,4.2), (0.54,0.44,0.63)>	<(68,5.7), (0.27,0.65,0.68)>	<(6.6,0.63), (0.3,0.22,0.63)>
particulate respirator ($A_{2}$) 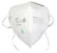	<(140,12.5), (0.54,0.55,0.49)>	<(40,3.7), (0.44,0.59,0.56)>	<(69,5.8), (0.61,0.48,0.54)>	<(9,1.1), (0.73,0.43,0.42)>
medical protective mask ($A_{3}$) 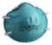	<(105,9), (0.53,0.48,0.29)>	<(36,3.3), (0.45,0.46,0.66)>	<(75,7.1), (0.73,0.55,0.44)>	<(7.5,0.72), (0.6,0.47,0.63)>
disposable medical mask ($A_{4}$) 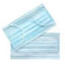	<(120,11), (0.73,0.48,0.29)>	<(35,3.2), (0.8,0.21,0.12)>	<(85,7.6), (0.28,0.55,0.44)>	<(8,0.9), (0.28,0.65,0.68)>
ordinary non-medical mask ($A_{5}$) 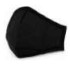	<(125,11.3), (0.39,0.58,0.64)>	<(45,4.3), (0.34,0.66,0.43)>	<(90,8.2), (0.45,0.68,0.31)>	<(7.2,0.71), (0.23,0.61,0.61)>
gas mask ($A_{6}$) 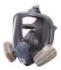	<(115,10.1), (0.1,0.7,0.25)>	<(37,3.4), (0.32,0.64,0.27)>	<(79,7.3), (0.43,0.65,0.37)>	<(8.3,0.82), (0.6,0.42,0.6)>

**Table 2:** Normalized decision information matrix.

	$C_{1}$	$C_{2}$	$C_{3}$	$C_{4}$
$A_{1}$	<(0.964,0.083), (0.29,0.54,0.61)>	<(1, 0.085), (0.54,0.44,0.63)>	<(0.756, 0.058), (0.27,0.65,0.68)>	<(0.733,0.05), (0.3,0.22,0.63)>
$A_{2}$	<(1, 0.089), (0.54,0.55,0.49)>	<(0.833, 0.08), (0.44,0.59,0.56)>	<(0.767, 0.059), (0.61,0.48,0.54)>	<(1,0.12), (0.73,0.43,0.42)>
$A_{3}$	<(0.75, 0.062), (0.53,0.48,0.29)>	<(0.75, 0.07), (0.45,0.46,0.66)>	<(0.838, 0.082), (0.73,0.55,0.44)>	<(0.833,0.06), (0.6,0.47,0.63)>
$A_{4}$	<(0.857, 0.081), (0.73,0.48,0.29)>	<(0.729, 0.068), (0.8,0.21,0.12)>	<(0.944, 0.083), (0.28,0.55,0.44)>	<(0.889, 0.09), (0.28,0.65,0.68)>
$A_{5}$	<(0.893, 0.082), (0.39,0.58,0.64)>	<(0.938, 0.096), (0.34,0.66,0.43)>	<(1, 0.091), (0.45,0.68,0.31)>	<(0.8,0.06), (0.23,0.61,0.61)>
$A_{6}$	<(0.821, 0.071), (0.1,0.7,0.25)>	<(0.771, 0.073), (0.32,0.64,0.27)>	<(0.878, 0.082), (0.43,0.65,0.37)>	<(0.922,0.07), (0.6,0.42,0.6)>

**Table 3:** The impact of parameters p,q

The Value of *p, q*	The Ranking Result
p,q = 0.3	$A_{4}>A_{3}>A_{6}>A_{2}>A_{1}>A_{5}$
p,q = 1	$A_{4}>A_{3}>A_{6}>A_{2}>A_{1}>A_{5}$
p,q = 7	$A_{4}>A_{3}>A_{2}>A_{6}>A_{1}>A_{5}$
p,q = 9	$A_{4}>A_{3}>A_{2}>A_{6}>A_{1}>A_{5}$
p = 0.1, q = 5	$A_{3}>A_{4}>A_{2}>A_{6}>A_{1}>A_{5}$
p = 5, q = 0.1	$A_{4}>A_{3}>A_{2}>A_{6}>A_{1}>A_{5}$
p = 1, q = 9	$A_{4}>A_{3}>A_{2}>A_{6}>A_{1}>A_{5}$
p = 9, q = 1	$A_{3}>A_{4}>A_{2}>A_{6}>A_{1}>A_{5}$

**Table 4:** The impact of criterion weight w on the ranking.

The Value of w	The Ranking Result
$w_{1}$ = 0.85, $w_{2}$ = $w_{3}$ = $w_{4}$ = 0.05	$A_{4}>A_{3}>A_{6}>A_{2}>A_{1}>A_{5}$
$w_{1}$ = 0.85, $w_{2}$ = $w_{3}$ = $w_{4}$ = 0.05	$A_{4}>A_{6}>A_{3}>A_{2}>A_{1}>A_{5}$
$w_{1}$ = 0.85, $w_{2}$ = $w_{3}$ = $w_{4}$ = 0.05	$A_{3}>A_{2}>A_{6}>A_{5}>A_{4}>A_{1}$
$w_{1}$ = 0.85, $w_{2}$ = $w_{3}$ = $w_{4}$ = 0.05	$A_{1}>A_{2}>A_{3}>A_{6}>A_{5}>A_{4}$

**Table 5:** The characteristic comparisons of different methods.

Methods	Information by SpFN	Information by NFN	Whether Considered the Interrelationships between Arguments
Yager [23]’s method based on PyFS	no	no	no
Cuong [29,30]’s method based on PtFS	no	no	no
Wang et al. [44]’s method based on INFN and entropy	no	yes	no
Yang et al. [47]’s method based on INFN	no	yes	no
Zhang et al. [48]’s method based on INFN and Heronian Mean Operator	no	yes	yes
The proposed method	yes	yes	yes

**Table 6:** Spearman’s rank-correlation test results.

Ranking Results								
	$A_{1}$	$A_{2}$	$A_{3}$	$A_{4}$	$A_{5}$	$A_{6}$		
The proposed method (P1)	5	4	2	1	6	3		
The method using Archimedean operator by [32] (P2)	5	4	3	1	6	2		
The method using logarithmic operation by [36] (P3)	5	3	2	1	6	4		
The method using cosine similarity measures by [37] (P4)	3	2	4	1	5	6		
The method using induced generalized aggregation operator by [45] (P5)	2	4	1	3	5	6		
	**Ranking Difference** $d_{i}$						**Spearman’s Test Results**	
							$r_{s}$	*Z*
P1-P2	0	0	1	0	0	1	0.943	2.108
P1-P3	0	1	0	0	0	1	0.943	2.108
P1-P4	2	2	2	0	1	3	0.371	0.831
P1-P5	3	0	1	2	1	3	0.314	0.703

## Appendix A

**Proof** **of Proposition 1.** Based on Definition 6, we found that (1), (3), (5), (6), and (7) are true, respectively. Further, (2), (4), and (8) need to be proved as follows: Let the NFN of $\left(A_{1}⊕A_{2}\right)⊕A_{3}$ and $A_{1}⊕\left(A_{2}⊕A_{3}\right)$ be $F_{\left(A_{1}⊕A_{2}\right)⊕A_{3}}$ and $F_{A_{1}⊕\left(A_{2}⊕A_{3}\right)}$, the PoMBD of them be $\delta_{\left(A_{1}⊕A_{2}\right)⊕A_{3}}$ and $\delta_{A_{1}⊕\left(A_{2}⊕A_{3}\right)}$, the NeuMBD of them be $\gamma_{\left(A_{1}⊕A_{2}\right)⊕A_{3}}$ and $\gamma_{A_{1}⊕\left(A_{2}⊕A_{3}\right)}$, the NegMBD of them be $\tau_{\left(A_{1}⊕A_{2}\right)⊕A_{3}}$, and $\tau_{A_{1}⊕\left(A_{2}⊕A_{3}\right)}$, respectively, then we can get: For (2), i.e., $\left(A_{1}⊕A_{2}\right)⊕A_{3}=A_{1}⊕\left(A_{2}⊕A_{3}\right)$, we have $F_{\left(A_{1}⊕A_{2}\right)⊕A_{3}}$ = $F_{A_{1}⊕\left(A_{2}⊕A_{3}\right)}$ = $\left(\alpha_{1}+\alpha_{2}+\alpha_{3},\sigma_{1}+\sigma_{2}+\sigma_{3}\right)$, $\begin{array}{c} \delta_{\left(A_{1}⊕A_{2}\right)⊕A_{3}}=\sqrt{\delta_{1}^{2}+\delta_{2}^{2}-\delta_{1}^{2}\delta_{2}^{2}+\delta_{3}^{2}-(\delta_{1}^{2}+\delta_{2}^{2}-\delta_{1}^{2}\delta_{2}^{2})\delta_{3}^{2}}\\ \;=\sqrt{\delta_{1}^{2}+\delta_{2}^{2}+\delta_{3}^{2}-\delta_{1}^{2}\delta_{2}^{2}-\delta_{1}^{2}\delta_{3}^{2}-\delta_{2}^{2}\delta_{3}^{2}+\delta_{1}^{2}\delta_{2}^{2}\delta_{3}^{2}} \end{array}$, $\begin{array}{c} \delta_{A_{1}⊕\left(A_{2}⊕A_{3}\right)}=\sqrt{\delta_{2}^{2}+\delta_{3}^{2}-\delta_{2}^{2}\delta_{3}^{2}+\delta_{1}^{2}-(\delta_{2}^{2}+\delta_{3}^{2}-\delta_{2}^{2}\delta_{3}^{2})\delta_{1}^{2}}\\ \;=\sqrt{{\left(\delta_{1}^{2}+\delta_{2}^{2}+\delta_{3}^{2}-\delta_{1}^{2}\delta_{2}^{2}-\delta_{1}^{2}\delta_{3}^{2}-\delta_{2}^{2}\delta_{3}^{2}+\delta_{1}^{2}\delta_{2}^{2}\delta_{3}^{2}\right)}^{1/q}} \end{array}$, and $\delta_{\left(A_{1}⊕A_{2}\right)⊕A_{3}}$ = $\delta_{A_{1}⊕\left(A_{2}⊕A_{3}\right)}$. Similarly, we can get $\gamma_{\left(A_{1}⊕A_{2}\right)⊕A_{3}}$ = $\gamma_{A_{1}⊕\left(A_{2}⊕A_{3}\right)}$, and $\tau_{\left(A_{1}⊕A_{2}\right)⊕A_{3}}$ = $\tau_{A_{1}⊕\left(A_{2}⊕A_{3}\right)}$. Therefore, $\left(A_{1}⊕A_{2}\right)⊕A_{3}=A_{1}⊕\left(A_{2}⊕A_{3}\right)$. For (4), i.e., $\left(A_{1}⊗A_{2}\right)⊗A_{3}=A_{1}⊗\left(A_{2}⊗A_{3}\right)$. $\begin{array}{l} F_{\left(A_{1}⊕A_{2}\right)⊕A_{3}}=F_{A_{1}⊕\left(A_{2}⊕A_{3}\right)}=\left(\alpha_{1}\alpha_{2}\alpha_{3},\alpha_{1}\alpha_{2}\alpha_{3}\sqrt{\left(\frac{\sigma_{1}^{2}}{\alpha_{1}^{2}}+\frac{\sigma_{1}^{2}}{\alpha_{2}^{2}}\right)+\frac{\sigma_{3}^{2}}{\alpha_{3}^{2}}}\right)\\ =\left(\alpha_{1}\alpha_{2}\alpha_{3},\alpha_{1}\alpha_{2}\alpha_{3}\sqrt{\frac{\sigma_{1}^{2}}{\alpha_{1}^{2}}+\left(\frac{\sigma_{1}^{2}}{\alpha_{2}^{2}}+\frac{\sigma_{3}^{2}}{\alpha_{3}^{2}}\right)}\right) \end{array}$, $\begin{array}{l} \gamma_{\left(A_{1}⊗A_{2}\right)⊗A_{3}}=\sqrt{\gamma_{1}^{2}+\gamma_{2}^{2}-\gamma_{1}^{2}\gamma_{2}^{2}+\gamma_{3}^{2}-(\gamma_{1}^{2}+\gamma_{2}^{2}-\gamma_{1}^{2}\gamma_{2}^{2})\gamma_{3}^{2}}\\ \;=\sqrt{\gamma_{1}^{2}+\gamma_{2}^{2}+\gamma_{3}^{2}-\gamma_{1}^{2}\gamma_{2}^{2}-\gamma_{1}^{2}\gamma_{3}^{2}-\gamma_{2}^{2}\gamma_{3}^{2}+\gamma_{1}^{2}\gamma_{2}^{2}\gamma_{3}^{2}} \end{array}$, $\begin{array}{l} \gamma_{A_{1}⊗\left(A_{2}⊗A_{3}\right)}=\sqrt{\gamma_{2}^{2}+\gamma_{3}^{2}-\gamma_{2}^{2}\gamma_{3}^{2}+\gamma_{1}^{2}-(\gamma_{2}^{2}+\gamma_{3}^{2}-\gamma_{2}^{2}\gamma_{3}^{2})\gamma_{1}^{2}}\\ =\sqrt{\gamma_{1}^{2}+\gamma_{2}^{2}+\gamma_{3}^{2}-\gamma_{1}^{2}\gamma_{2}^{2}-\gamma_{1}^{2}\gamma_{3}^{2}-\gamma_{2}^{2}\gamma_{3}^{2}+\gamma_{1}^{2}\gamma_{2}^{2}\gamma_{3}^{2}} \end{array}$, and $\gamma_{\left(A_{1}⊕A_{2}\right)⊕A_{3}}$ = $\gamma_{A_{1}⊕\left(A_{2}⊕A_{3}\right)}$. Similarly, we get $\tau_{\left(A_{1}⊕A_{2}\right)⊕A_{3}}$ = $\tau_{A_{1}⊕\left(A_{2}⊕A_{3}\right)}$, and $\delta_{\left(A_{1}⊕A_{2}\right)⊕A_{3}}$ = $\delta_{A_{1}⊕\left(A_{2}⊕A_{3}\right)}$. Therefore, $\left(A_{1}⊗A_{2}\right)⊗A_{3}=A_{1}⊗\left(A_{2}⊗A_{3}\right)$. We now prove item (8), i.e., $A_{1}^{\lambda_{1}}⊗A_{1}^{\lambda_{2}}=A_{1}^{\lambda_{1}+\lambda_{2}}$. Let $A_{1}=\langle (\alpha_{1},\sigma_{1}),\left(\delta_{1},\gamma_{1},\tau_{1}\right)\rangle$ be a SpNoFN, $\lambda_{1},\lambda_{1}\geq 0$, and the NFN of $A_{1}^{\lambda_{1}}⊗A_{1}^{\lambda_{2}}$ and $A_{1}^{\lambda_{1}+\lambda_{2}}$ be $F_{A_{1}^{\lambda_{1}}⊗A_{1}^{\lambda_{2}}}$ and $F_{A_{1}^{\lambda_{1}+\lambda_{2}}}$. We can obtain that $\begin{array}{l} F_{A_{1}^{\lambda_{1}}⊗A_{1}^{\lambda_{2}}}=F_{A_{1}^{\lambda_{1}+\lambda_{2}}}\\ =\left(\alpha_{1}^{\lambda_{1}}\alpha_{1}^{\lambda_{2}},\alpha_{1}^{\lambda_{1}}\alpha_{1}^{\lambda_{2}}\sqrt{\lambda_{1}\alpha_{1}^{-2}\sigma_{1}^{2}+\lambda_{2}\alpha_{1}^{-2}\sigma_{1}^{2}}\right)\\ =\left(\alpha_{1}^{{\left(\lambda_{1}+\lambda \right)}_{2}},\alpha_{1}^{{\left(\lambda_{1}+\lambda \right)}_{2}}\frac{\sigma_{1}^{}}{\alpha_{1}^{}}\sqrt{\left(\lambda_{1}+\lambda_{2}\right)}\right) \end{array}$, and $\begin{array}{l} \delta_{1}^{\lambda_{1}}⊗\delta_{1}^{\lambda_{2}}=\sqrt{1-{\left(1-\delta_{1}^{2}\right)}^{\lambda_{1}}+1-{\left(1-\delta_{1}^{2}\right)}^{\lambda_{2}}-\left(\left(1-{\left(1-\delta_{1}^{2}\right)}^{\lambda_{1}}\right)\left(1-{\left(1-\delta_{1}^{2}\right)}^{\lambda_{2}}\right)\right)}\\ =\sqrt{1-{\left(1-\delta_{1}^{2}\right)}^{\lambda_{1}}+1-{\left(1-\delta_{1}^{2}\right)}^{\lambda_{2}}-\left(\left(1-{\left(1-\delta_{1}^{2}\right)}^{\lambda_{1}}\right)\left(1-{\left(1-\delta_{1}^{2}\right)}^{\lambda_{2}}\right)\right)}\\ =\sqrt{1-{\left(1-\delta_{1}^{2}\right)}^{\lambda_{1}}+\left(1-{\left(1-\delta_{1}^{2}\right)}^{\lambda_{2}}\right){\left(1-\delta_{1}^{2}\right)}^{\lambda_{1}}}\\ =\sqrt{1-{\left(1-\delta_{1}^{2}\right)}^{\lambda_{1}+\lambda_{2}}}=\delta_{1}^{\left(\lambda_{1}+\lambda_{2}\right)} \end{array}$. Similarly, we get $\gamma_{1}^{\lambda_{1}}⊗\gamma_{1}^{\lambda_{2}}=\gamma_{1}^{\left(\lambda_{1}+\lambda_{2}\right)}$, and $\tau_{1}^{\lambda_{1}}⊗\tau_{1}^{\lambda_{2}}=\tau_{1}^{\left(\lambda_{1}+\lambda_{2}\right)}$. As such, $A_{1}^{\lambda_{1}}⊗A_{1}^{\lambda_{2}}=A_{1}^{\lambda_{1}+\lambda_{2}}$ is true. □

## Appendix B

**Proof** **of Theorem 1.** According to the operations of SpNoFNs, we can get: $A_{i}^{p}=\left[\left(\alpha_{i}^{p},p^{\frac{1}{2}}\alpha_{i}^{p-1}\sigma_{i}\right),\left(\delta_{i}^{p},\sqrt{1-{\left(1-\gamma_{i}^{2}\right)}^{p}},\sqrt{1-{\left(1-\tau_{i}^{2}\right)}^{p}}\right)\right]$, $A_{j}^{q}=\left[\left(\alpha_{j}^{q},q^{\frac{1}{2}}\alpha_{i}^{q-1}\sigma_{i}\right),\left(\delta_{j}^{q},\sqrt{1-{\left(1-\gamma_{i}^{2}\right)}^{q}},\sqrt{1-{\left(1-\tau_{i}^{2}\right)}^{q}}\right)\right]$, and

$$\begin{array}{l} {\left(A_{i}\right)}^{p}⊗{\left(A_{j}\right)}^{q}\\ =\left[\begin{array}{l} \left(\alpha_{i}^{p}\alpha_{j}^{q},\alpha_{i}^{p}\alpha_{j}^{q}\sqrt{\frac{p\alpha_{i}^{2\left(p-1\right)}\sigma_{i}^{2}}{\alpha_{i}^{2p}}+\frac{q\alpha_{j}^{2\left(q-1\right)}\sigma_{j}^{2}}{\alpha_{j}^{2q}}}\right),\\ \left(\begin{array}{l} \delta_{i}^{p}\delta_{j}^{q},\\ \sqrt{\left(1-{\left(1-\gamma_{i}^{2}\right)}^{p}\right)+\left(1-{\left(1-\gamma_{j}^{2}\right)}^{q}\right)-\left(1-{\left(1-\gamma_{i}^{2}\right)}^{p}\right)\left(1-{\left(1-\gamma_{j}^{2}\right)}^{q}\right)},\\ \sqrt{\left(1-{\left(1-\tau_{i}^{2}\right)}^{p}\right)+\left(1-{\left(1-\tau_{j}^{2}\right)}^{q}\right)-\left(1-{\left(1-\tau_{i}^{2}\right)}^{p}\right)\left(1-{\left(1-\tau_{j}^{2}\right)}^{q}\right)} \end{array}\right) \end{array}\right]\\ =\left[\begin{array}{l} \left(\alpha_{i}^{p}\alpha_{j}^{q},\alpha_{i}^{p}\alpha_{j}^{q}\sqrt{\frac{p\sigma_{i}^{2}}{\alpha_{i}^{2}}+\frac{q\sigma_{j}^{2}}{\alpha_{j}^{2}}}\right),\\ \left(\delta_{i}^{p}\delta_{j}^{q},\sqrt{1-{\left(1-\gamma_{i}^{2}\right)}^{p}{\left(1-\gamma_{j}^{2}\right)}^{q}},\sqrt{1-{\left(1-\tau_{i}^{2}\right)}^{p}{\left(1-\tau_{j}^{2}\right)}^{q}}\right) \end{array}\right] \end{array}$$

The mathematical induction method is used to obtain

$$\sum_{i=1}^{n}{\left(A_{i}\right)}^{p}⊗{\left(A_{j}\right)}^{q}=\left[\begin{array}{c} \sum_{i=1}^{n}\alpha_{i}^{p}\alpha_{j}^{q},\sum_{i=1}^{n}\alpha_{i}^{p}\alpha_{j}^{q}\sqrt{\frac{p\sigma_{i}^{2}}{\alpha_{i}^{2}}+\frac{q\sigma_{j}^{2}}{\alpha_{j}^{2}}}\sqrt{1-\prod_{i=1}^{n}\left(1-{\left(\delta_{i}^{p}\delta_{j}^{q}\right)}^{2}\right)},\\ \prod_{i=1}^{n}\sqrt{\left(1-{\left(1-\gamma_{i}^{2}\right)}^{p}{\left(1-\gamma_{j}^{2}\right)}^{q}\right)},\prod_{i=1}^{n}\sqrt{\left(1-{\left(1-\tau_{i}^{2}\right)}^{p}{\left(1-\tau_{j}^{2}\right)}^{q}\right)} \end{array}\right],$$

and

$$\sum_{\begin{array}{c} i,j=1\\ i\neq j \end{array}}^{n}{\left(A_{i}\right)}^{p}⊗{\left(A_{j}\right)}^{q}=\left[\begin{array}{l} \sum_{\begin{array}{c} i,j=1\\ i\neq j \end{array}}^{n}\alpha_{i}^{p}\alpha_{j}^{q},\sum_{\begin{array}{c} i,j=1\\ i\neq j \end{array}}^{n}\alpha_{i}^{p}\alpha_{j}^{q}\sqrt{\frac{p\sigma_{i}^{2}}{\alpha_{i}^{2}}+\frac{q\sigma_{j}^{2}}{\alpha_{j}^{2}}}\sqrt{1-\prod_{\begin{array}{c} i,j=1\\ i\neq j \end{array}}^{n}\left(1-{\left(\delta_{i}^{p}\delta_{j}^{q}\right)}^{2}\right)},\\ \prod_{\begin{array}{c} i,j=1\\ i\neq j \end{array}}^{n}\sqrt{\left(1-{\left(1-\gamma_{i}^{2}\right)}^{p}{\left(1-\gamma_{j}^{2}\right)}^{q}\right)},\prod_{\begin{array}{c} i,j=1\\ i\neq j \end{array}}^{n}\sqrt{\left(1-{\left(1-\tau_{i}^{2}\right)}^{p}{\left(1-\tau_{j}^{2}\right)}^{q}\right)} \end{array}\right].$$

Furthermore, the following result can be derived:

$$\begin{array}{l} \frac{1}{n(n-1)}\sum_{\begin{array}{c} i,j=1\\ i\neq j \end{array}}^{n}{\left(A_{i}\right)}^{p}⊗{\left(A_{j}\right)}^{q}\\ =\left[\begin{array}{l} \left(\frac{1}{n(n-1)}\sum_{\begin{array}{c} i,j=1\\ i\neq j \end{array}}^{n}\alpha_{i}^{p}\alpha_{j}^{q},\frac{1}{n(n-1)}\sum_{\begin{array}{c} i,j=1\\ i\neq j \end{array}}^{n}\alpha_{i}^{p}\alpha_{j}^{q}\sqrt{\frac{p\sigma_{i}^{2}}{\alpha_{i}^{2}}+\frac{q\sigma_{j}^{2}}{\alpha_{j}^{2}}}\right),\\ \left(\begin{array}{l} \sqrt{1-{\left(\prod_{\begin{array}{c} i,j=1\\ i\neq j \end{array}}^{n}\left(1-{\left(\delta_{i}^{p}\delta_{j}^{q}\right)}^{2}\right)\right)}^{\frac{1}{n(n-1)}}},{\left(\prod_{\begin{array}{c} i,j=1\\ i\neq j \end{array}}^{n}\sqrt{\left(1-{\left(1-\gamma_{i}^{2}\right)}^{p}{\left(1-\gamma_{j}^{2}\right)}^{q}\right)}\right)}^{\frac{1}{n(n-1)}},\\ {\left(\prod_{\begin{array}{c} i,j=1\\ i\neq j \end{array}}^{n}\sqrt{\left(1-{\left(1-\tau_{i}^{2}\right)}^{p}{\left(1-\tau_{j}^{2}\right)}^{q}\right)}\right)}^{\frac{1}{n(n-1)}} \end{array}\right) \end{array}\right], \end{array}$$

and

$$\begin{array}{l} {\left(\frac{1}{n(n-1)}\sum_{\begin{array}{c} i,j=1\\ i\neq j \end{array}}^{n}{\left(A_{i}\right)}^{p}⊗{\left(A_{j}\right)}^{q}\right)}^{\frac{1}{p+q}}\\ =\left[\begin{array}{l} \left({\left(\frac{1}{n(n-1)}\sum_{\begin{array}{c} i,j=1\\ i\neq j \end{array}}^{n}\alpha_{i}^{p}\alpha_{j}^{q}\right)}^{\frac{1}{p+q}},\sqrt{\frac{1}{p+q}}{\left(\frac{1}{n(n-1)}\sum_{\begin{array}{c} i,j=1\\ i\neq j \end{array}}^{n}\alpha_{i}^{p}\alpha_{j}^{q}\right)}^{\frac{1}{p+q}-1}\left(\frac{1}{n(n-1)}\sum_{\begin{array}{c} i,j=1\\ i\neq j \end{array}}^{n}\alpha_{i}^{p}\alpha_{j}^{q}\sqrt{\frac{p\sigma_{i}^{2}}{\alpha_{i}^{2}}+\frac{q\sigma_{j}^{2}}{\alpha_{j}^{2}}}\right)\right),\\ \left(\begin{array}{l} {\left(\sqrt{1-{\left(\prod_{\begin{array}{c} i,j=1\\ i\neq j \end{array}}^{n}\left(1-{\left(\delta_{i}^{p}\delta_{j}^{q}\right)}^{2}\right)\right)}^{\frac{1}{n(n-1)}}}\right)}^{\frac{1}{p+q}},\sqrt{1-{\left(1-{\left(\prod_{\begin{array}{c} ,j=1\\ i\neq j \end{array}}^{n}\left(1-{\left(1-\gamma_{i}^{2}\right)}^{p}{\left(1-\gamma_{j}^{2}\right)}^{q}\right)\right)}^{\frac{1}{n(n-1)}}\right)}^{\frac{1}{p+q}}},\\ \sqrt{1-{\left(1-{\left(\prod_{\begin{array}{c} ,j=1\\ i\neq j \end{array}}^{n}\left(1-{\left(1-\tau_{i}^{2}\right)}^{p}{\left(1-\tau_{j}^{2}\right)}^{q}\right)\right)}^{\frac{1}{n(n-1)}}\right)}^{\frac{1}{p+q}}} \end{array}\right) \end{array}\right]. \end{array}$$

The Proof is completed. □

## Appendix C

**Proof** **of Theorem 2.**

$$\begin{array}{l} SpNoFBM\left(A_{1},A_{2},\cdots ,A_{n}\right)\\ ={\left(\frac{1}{n(n-1)}\sum_{\begin{array}{c} i,j=1\\ i\neq j \end{array}}^{n}{\left(A_{i}\right)}^{p}⊗{\left(A_{j}\right)}^{q}\right)}^{\frac{1}{p+q}}\\ ={\left(\frac{1}{n(n-1)}\sum_{\begin{array}{c} i,j=1\\ i\neq j \end{array}}^{n}{\left(A_{i}\right)}^{p}⊗{\left(A_{j}\right)}^{q}\right)}^{\frac{1}{p+q}}\\ ={\left(\frac{1}{n(n-1)}\sum_{\begin{array}{c} i,j=1\\ i\neq j \end{array}}^{n}{\left(A\right)}^{p+q}\right)}^{\frac{1}{p+q}}=A \end{array}$$

 □

**Proof** **of Theorem 3.**

$$\begin{array}{c} SpNoFBM\left(A_{1},A_{2},\cdots ,A_{n}\right)\\ ={\left(\frac{1}{n(n-1)}\sum_{\begin{array}{c} i,j=1\\ i\neq j \end{array}}^{n}{\left(A_{i}\right)}^{p}⊗{\left(A_{j}\right)}^{q}\right)}^{\frac{1}{p+q}}\\ \leq {\left(\frac{1}{n(n-1)}\sum_{\begin{array}{c} i,j=1\\ i\neq j \end{array}}^{n}{\left(A^{+}\right)}^{p}⊗{\left(A^{+}\right)}^{q}\right)}^{\frac{1}{p+q}}\\ \leq {\left(\frac{1}{n(n-1)}\sum_{\begin{array}{c} i,j=1\\ i\neq j \end{array}}^{n}{\left(A^{+}\right)}^{p}{}^{+q}\right)}^{\frac{1}{p+q}}=A^{+} \end{array}.$$

Similarly, we can get $A^{-}\leq SpNoFBM\left(A_{1},A_{2},\cdots ,A_{n}\right)$. Therefore, $A^{-}\leq SpNoFBM\left(A_{1},A_{2},\cdots ,A_{n}\right)\leq A^{+}$. □

**Proof** **of Theorem 4.** For ∀i, there exist $\alpha_{A_{i}}\leq \alpha_{B_{i}}$ and $\delta_{A_{i}}\leq \delta_{B_{i}},\gamma_{A_{i}}\geq \gamma_{B_{i}},\tau_{A_{i}}\geq \tau_{B_{i}}$. Then, ${\left(\frac{1}{n(n-1)}\sum_{\begin{array}{c} i,j=1\\ i\neq j \end{array}}^{n}\alpha_{A_{i}}^{p}\alpha_{A_{j}}^{q}\right)}^{\frac{1}{p+q}}\leq {\left(\frac{1}{n(n-1)}\sum_{\begin{array}{c} i,j=1\\ i\neq j \end{array}}^{n}\alpha_{B_{i}}^{p}\alpha_{B_{j}}^{q}\right)}^{\frac{1}{p+q}}$,
$\begin{array}{l} {\left(\sqrt{1-{\left(\prod_{\begin{array}{l} i,j=1\\ i\neq j \end{array}}^{n}\left(\text{1}-{\left(\delta_{A_{i}}^{p}\delta_{A_{i}}^{q}\right)}^{2}\right)\right)}^{\frac{1}{n(n-1)}}}\right)}^{\frac{1}{p+q}}\leq {\left(\sqrt{1-{\left(\prod_{\begin{array}{l} i,j=1\\ i\neq j \end{array}}^{n}\left(\text{1}-{\left(\delta_{B_{i}}^{p}\delta_{B_{i}}^{q}\right)}^{2}\right)\right)}^{\frac{1}{n(n-1)}}}\right)}^{\frac{1}{p+q}},\\ \sqrt{1-{\left(1-{\left(\prod_{\begin{array}{l} ,j=1\\ i\neq j \end{array}}^{n}\left(1-{\left(1-\gamma_{A_{i}}^{2}\right)}^{p}{\left(1-\gamma_{A_{i}}^{2}\right)}^{q}\right)\right)}^{\frac{\text{1}}{n(n-1)}}\right)}^{\frac{1}{p+q}}}\geq \sqrt{1-{\left(1-{\left(\prod_{\begin{array}{l} ,j=1\\ i\neq j \end{array}}^{n}\left(1-{\left(1-\gamma_{B_{i}}^{2}\right)}^{p}{\left(1-\gamma_{B_{i}}^{2}\right)}^{q}\right)\right)}^{\frac{\text{1}}{n(n-1)}}\right)}^{\frac{1}{p+q}}}, \end{array}$
and $\sqrt{1-{\left(1-{\left(\prod_{\begin{array}{c} ,j=1\\ i\neq j \end{array}}^{n}\left(1-{\left(1-\tau_{A_{i}}^{2}\right)}^{p}{\left(1-\tau_{A_{i}}^{2}\right)}^{q}\right)\right)}^{\frac{1}{n(n-1)}}\right)}^{\frac{1}{p+q}}}\geq \sqrt{1-{\left(1-{\left(\prod_{\begin{array}{c} ,j=1\\ i\neq j \end{array}}^{n}\left(1-{\left(1-\tau_{B_{i}}^{2}\right)}^{p}{\left(1-\tau_{B_{i}}^{2}\right)}^{q}\right)\right)}^{\frac{1}{n(n-1)}}\right)}^{\frac{1}{p+q}}}$. Based on the score function in Definition 7, we can get $SpNoFBM\left(A_{1},A_{2},\cdots ,A_{n}\right)\leq SpNoFBM\left(B_{1},B_{2},\cdots ,B_{n}\right)$. □

## Appendix D

**Proof** **of Theorem 5.** According to the operational rules of SpNoFNs, we get $A_{i}w_{i}=\langle \left(\alpha_{i}w_{i},\sigma_{i}w_{i}\right),\left(\sqrt{1-{\left(1-\delta_{i}^{2}\right)}^{w_{i}}},\gamma_{i}^{w_{i}},\tau_{i}^{w_{i}}\right)\rangle$. Hence, for real *p, q > 0,* we have ${\left(A_{i}w_{i}\right)}^{p}=\langle \begin{array}{c} \left({\left(\alpha_{i}w_{i}\right)}^{p},p^{\frac{1}{2}}{\left(\alpha_{i}w_{i}\right)}^{p-1}\left(\sigma_{i}w_{i}\right)\right),\\ \left({\left(\sqrt{1-{\left(1-\delta_{i}^{2}\right)}^{w_{i}}}\right)}^{p},\sqrt{1-{\left(1-{\left(\gamma_{i}^{w_{i}}\right)}^{2}\right)}^{p}},\sqrt{1-{\left(1-{\left(\tau_{i}^{w_{i}}\right)}^{2}\right)}^{p}}\right) \end{array}\rangle$, ${\left(A_{j}w_{j}\right)}^{q}=\langle \begin{array}{c} \left({\left(\alpha_{j}w_{j}\right)}^{q},q^{\frac{1}{2}}{\left(\alpha_{j}w_{j}\right)}^{q-1}\left(\sigma_{j}w_{j}\right)\right),\\ \left({\left(\sqrt{1-{\left(1-\delta_{j}^{2}\right)}^{w_{j}}}\right)}^{q},\sqrt{1-{\left(1-{\left(\gamma_{j}^{w_{j}}\right)}^{2}\right)}^{q}},\sqrt{1-{\left(1-{\left(\tau_{j}^{w_{j}}\right)}^{2}\right)}^{q}}\right) \end{array}\rangle$, and

$$\begin{array}{l} {\left(A_{i}w_{i}\right)}^{p}⊗{\left(A_{j}w_{j}\right)}^{q}\\ =\left[\begin{array}{l} \left({\left(\alpha_{i}w_{i}\right)}^{p}{\left(\alpha_{j}w_{j}\right)}^{q},{\left(\alpha_{i}w_{i}\right)}^{p}{\left(\alpha_{j}w_{j}\right)}^{q}\sqrt{\frac{p{\left(\alpha_{i}w_{i}\right)}^{2\left(p-1\right)}{\left(\sigma_{i}w_{i}\right)}^{2}}{{\left(\alpha_{i}w_{i}\right)}^{2p}}+\frac{q{\left(\alpha_{j}w_{j}\right)}^{2\left(q-1\right)}{\left(\sigma_{j}w_{j}\right)}^{2}}{{\left(\alpha_{j}w_{j}\right)}^{2q}}}\right),\\ \left(\begin{array}{l} {\left(\sqrt{1-{\left(1-\delta_{i}^{2}\right)}^{w_{i}}}\right)}^{p}{\left(\sqrt{1-{\left(1-\delta_{j}^{2}\right)}^{w_{j}}}\right)}^{q},\\ \sqrt{{\left(\sqrt{1-{\left(1-{\left(\gamma_{i}^{w_{i}}\right)}^{2}\right)}^{p}}\right)}^{2}+{\left(\sqrt{1-{\left(1-{\left(\gamma_{j}^{w_{j}}\right)}^{2}\right)}^{q}}\right)}^{2}-{\left(\sqrt{1-{\left(1-{\left(\gamma_{i}^{w_{i}}\right)}^{2}\right)}^{p}}\right)}^{2}{\left(\sqrt{1-{\left(1-{\left(\gamma_{j}^{w_{j}}\right)}^{2}\right)}^{q}}\right)}^{2}},\\ \sqrt{{\left(\sqrt{1-{\left(1-{\left(\tau_{i}^{w_{i}}\right)}^{2}\right)}^{p}}\right)}^{2}+{\left(\sqrt{1-{\left(1-{\left(\tau_{j}^{w_{j}}\right)}^{2}\right)}^{q}}\right)}^{2}-{\left(\sqrt{1-{\left(1-{\left(\tau_{i}^{w_{i}}\right)}^{2}\right)}^{p}}\right)}^{2}{\left(\sqrt{1-{\left(1-{\left(\tau_{j}^{w_{j}}\right)}^{2}\right)}^{q}}\right)}^{2}} \end{array}\right) \end{array}\right]\\ =\left[\begin{array}{l} \left({\left(\alpha_{i}w_{i}\right)}^{p}{\left(\alpha_{j}w_{j}\right)}^{q},{\left(\alpha_{i}w_{i}\right)}^{p}{\left(\alpha_{j}w_{j}\right)}^{q}\sqrt{\frac{p{\left(\sigma_{i}w_{i}\right)}^{2}}{{\left(\alpha_{i}w_{i}\right)}^{2}}+\frac{q{\left(\sigma_{j}w_{j}\right)}^{2}}{{\left(\alpha_{j}w_{j}\right)}^{2}}}\right),\\ \left(\begin{array}{l} {\left(\sqrt{1-{\left(1-\delta_{i}^{2}\right)}^{w_{i}}}\right)}^{p}{\left(\sqrt{1-{\left(1-\delta_{j}^{2}\right)}^{w_{j}}}\right)}^{q},\\ \sqrt{1-\left(1-{\left(\sqrt{1-{\left(1-{\left(\gamma_{i}^{w_{i}}\right)}^{2}\right)}^{p}}\right)}^{2}\right)\left(1-{\left(\sqrt{1-{\left(1-{\left(\gamma_{j}^{w_{j}}\right)}^{2}\right)}^{q}}\right)}^{2}\right)},\\ \sqrt{1-\left(1-{\left(\sqrt{1-{\left(1-{\left(\tau_{i}^{w_{i}}\right)}^{2}\right)}^{p}}\right)}^{2}\right)\left(1-{\left(\sqrt{1-{\left(1-{\left(\tau_{j}^{w_{j}}\right)}^{2}\right)}^{q}}\right)}^{2}\right)} \end{array}\right) \end{array}\right]\\ =\left[\begin{array}{l} \left({\left(\alpha_{i}w_{i}\right)}^{p}{\left(\alpha_{j}w_{j}\right)}^{q},{\left(\alpha_{i}w_{i}\right)}^{p}{\left(\alpha_{j}w_{j}\right)}^{q}\sqrt{\frac{p{\left(\sigma_{i}w_{i}\right)}^{2}}{{\left(\alpha_{i}w_{i}\right)}^{2}}+\frac{q{\left(\sigma_{j}w_{j}\right)}^{2}}{{\left(\alpha_{j}w_{j}\right)}^{2}}}\right),\\ \left(\begin{array}{l} {\left(\sqrt{1-{\left(1-\delta_{i}^{2}\right)}^{w_{i}}}\right)}^{p}{\left(\sqrt{1-{\left(1-\delta_{j}^{2}\right)}^{w_{j}}}\right)}^{q},\\ \sqrt{1-{\left(1-{\left(\gamma_{i}^{w_{i}}\right)}^{2}\right)}^{p}{\left(1-{\left(\gamma_{j}^{w_{j}}\right)}^{2}\right)}^{q}},\sqrt{1-{\left(1-{\left(\tau_{i}^{w_{i}}\right)}^{2}\right)}^{p}{\left(1-{\left(\tau_{j}^{w_{j}}\right)}^{2}\right)}^{q}} \end{array}\right) \end{array}\right] \end{array}$$

By using the mathematical induction method, we obtain

$$\begin{array}{l} \sum_{i=1}^{n}{\left(A_{i}w_{i}\right)}^{p}⊗{\left(A_{j}w_{j}\right)}^{q}\\ =\left[\begin{array}{l} \left(\sum_{i=1}^{n}{\left(\alpha_{i}w_{i}\right)}^{p}{\left(\alpha_{j}w_{j}\right)}^{q},\sum_{i=1}^{n}{\left(\alpha_{i}w_{i}\right)}^{p}{\left(\alpha_{j}w_{j}\right)}^{q}\sqrt{\frac{p{\left(\sigma_{i}w_{i}\right)}^{2}}{{\left(\alpha_{i}w_{i}\right)}^{2}}+\frac{q{\left(\sigma_{j}w_{j}\right)}^{2}}{{\left(\alpha_{j}w_{j}\right)}^{2}}}\right)\\ \sqrt{1-\prod_{i=1}^{n}\left(1-{\left(1-{\left(1-\delta_{i}^{2}\right)}^{w_{i}}\right)}^{p}{\left(1-{\left(1-\delta_{j}^{2}\right)}^{w_{j}}\right)}^{q}\right)},\prod_{i=1}^{n}\sqrt{1-{\left(1-{\left(\gamma_{i}^{w_{i}}\right)}^{2}\right)}^{p}}\sqrt{{\left(1-{\left(\gamma_{j}^{w_{j}}\right)}^{2}\right)}^{q}},\\ \prod_{i=1}^{n}\sqrt{1-{\left(1-{\left(\tau_{i}^{w_{i}}\right)}^{2}\right)}^{p}}\sqrt{{\left(1-{\left(\tau_{j}^{w_{j}}\right)}^{2}\right)}^{q}} \end{array}\right]\\ \sum_{\begin{array}{c} i,j=1\\ i\neq j \end{array}}^{n}{\left(A_{i}w_{i}\right)}^{p}⊗{\left(A_{j}w_{j}\right)}^{q}\\ =\left[\begin{array}{l} \left(\sum_{\begin{array}{c} i,j=1\\ i\neq j \end{array}}^{n}{\left(\alpha_{i}w_{i}\right)}^{p}{\left(\alpha_{j}w_{j}\right)}^{q},\sum_{\begin{array}{c} i,j=1\\ i\neq j \end{array}}^{n}{\left(\alpha_{i}w_{i}\right)}^{p}{\left(\alpha_{j}w_{j}\right)}^{q}\sqrt{\frac{p{\left(\sigma_{i}w_{i}\right)}^{2}}{{\left(\alpha_{i}w_{i}\right)}^{2}}+\frac{q{\left(\sigma_{j}w_{j}\right)}^{2}}{{\left(\alpha_{j}w_{j}\right)}^{2}}}\right)\\ \sqrt{1-\prod_{\begin{array}{c} i,j=1\\ i\neq j \end{array}}^{n}\left(1-{\left(1-{\left(1-\delta_{i}^{2}\right)}^{w_{i}}\right)}^{p}{\left(1-{\left(1-\delta_{j}^{2}\right)}^{w_{j}}\right)}^{q}\right)},\prod_{\begin{array}{c} i,j=1\\ i\neq j \end{array}}^{n}\sqrt{1-{\left(1-{\left(\gamma_{i}^{w_{2}}\right)}^{2}\right)}^{p}}\sqrt{1-{\left(1-{\left(\gamma_{j}^{w_{j}}\right)}^{2}\right)}^{q}},\\ \prod_{\begin{array}{c} i,j=1\\ i\neq j \end{array}}^{n}\sqrt{1-{\left(1-{\left(\tau_{i}^{w_{2}}\right)}^{2}\right)}^{p}}\sqrt{1-{\left(1-{\left(\tau_{j}^{w_{j}}\right)}^{2}\right)}^{q}} \end{array}\right] \end{array}$$

Furthermore, we can also get

$$\begin{array}{l} \frac{1}{n(n-1)}\sum_{\begin{array}{c} i,j=1\\ i\neq j \end{array}}^{n}{\left(A_{i}w_{i}\right)}^{p}⊗{\left(A_{j}w_{j}\right)}^{q}\\ =\left[\begin{array}{c} \left(\left(\frac{1}{n(n-1)}\sum_{\begin{array}{c} i,j=1\\ i\neq j \end{array}}^{n}{\left(\alpha_{i}w_{i}\right)}^{p}{\left(\alpha_{j}w_{j}\right)}^{q},\frac{1}{n(n-1)}\sum_{\begin{array}{c} i,j=1\\ i\neq j \end{array}}^{n}{\left(\alpha_{i}w_{i}\right)}^{p}{\left(\alpha_{j}w_{j}\right)}^{q}\sqrt{\frac{p{\left(\sigma_{i}w_{i}\right)}^{2}}{{\left(\alpha_{i}w_{i}\right)}^{2}}+\frac{q{\left(\sigma_{j}w_{j}\right)}^{2}}{{\left(\alpha_{j}w_{j}\right)}^{2}}}\right)\right),\\ \sqrt{1-{\left(\prod_{\begin{array}{c} i,j=1\\ i\neq j \end{array}}^{n}\left(1-{\left(1-{\left(1-\delta_{i}^{2}\right)}^{w_{i}}\right)}^{p}{\left(1-{\left(1-\delta_{j}^{2}\right)}^{w_{j}}\right)}^{q}\right)\right)}^{\frac{1}{n(n-1)}}},{\left(\prod_{\begin{array}{c} i,j=1\\ i\neq j \end{array}}^{n}\sqrt{1-{\left(1-{\left(\gamma_{i}^{w_{2}}\right)}^{2}\right)}^{p}}\sqrt{1-{\left(1-{\left(\gamma_{j}^{w_{j}}\right)}^{2}\right)}^{q}}\right)}^{\frac{1}{n(n-1)}},\\ {\left(\prod_{\begin{array}{c} i,j=1\\ i\neq j \end{array}}^{n}\sqrt{1-{\left(1-{\left(\tau_{i}^{w_{2}}\right)}^{2}\right)}^{p}}\sqrt{1-{\left(1-{\left(\tau_{j}^{w_{j}}\right)}^{2}\right)}^{q}}\right)}^{\frac{1}{n(n-1)}} \end{array}\right] \end{array}$$

$$\begin{array}{l} {\left(\frac{1}{n(n-1)}\sum_{\begin{array}{c} i,j=1\\ i\neq j \end{array}}^{n}{\left(A_{i}w_{i}\right)}^{p}⊗{\left(A_{j}w_{j}\right)}^{q}\right)}^{\frac{1}{p+q}}\\ =\left[\begin{array}{l} \left(\begin{array}{l} {\left(\frac{1}{n(n-1)}\sum_{\begin{array}{c} i,j=1\\ i\neq j \end{array}}^{n}{\left(\alpha_{i}w_{i}\right)}^{p}{\left(\alpha_{j}w_{j}\right)}^{q}\right)}^{\frac{1}{p+q}},\sqrt{\frac{1}{p+q}}{\left(\frac{1}{n(n-1)}\sum_{\begin{array}{c} i,j=1\\ i\neq j \end{array}}^{n}{\left(\alpha_{i}w_{i}\right)}^{p}{\left(\alpha_{j}w_{j}\right)}^{q}\right)}^{\frac{1}{p+q}-1}\cdot \\ \frac{1}{n(n-1)}\sum_{\begin{array}{c} i,j=1\\ i\neq j \end{array}}^{n}{\left(\alpha_{i}w_{i}\right)}^{p}{\left(\alpha_{j}w_{j}\right)}^{q}\sqrt{\frac{p{\left(\sigma_{i}w_{i}\right)}^{2}}{{\left(\alpha_{i}w_{i}\right)}^{2}}+\frac{q{\left(\sigma_{j}w_{j}\right)}^{2}}{{\left(\alpha_{j}w_{j}\right)}^{2}}} \end{array}\right),\\ \left(\begin{array}{l} {\left(\sqrt{1-{\left(\prod_{\begin{array}{c} i,j=1\\ i\neq j \end{array}}^{n}\left(1-{\left(1-{\left(1-\delta_{i}^{2}\right)}^{w_{i}}\right)}^{p}{\left(1-{\left(1-\delta_{j}^{2}\right)}^{w_{j}}\right)}^{q}\right)\right)}^{\frac{1}{n(n-1)}}}\right)}^{\frac{1}{p+q}},\\ \sqrt{1-{\left(1-{\left(\prod_{\begin{array}{c} i,j=1\\ i\neq j \end{array}}^{n}\left(1-{\left(1-{\left(\gamma_{i}^{w_{i}}\right)}^{2}\right)}^{p}\right)\left(1-{\left(1-{\left(\gamma_{j}^{w_{j}}\right)}^{2}\right)}^{q}\right)\right)}^{\frac{1}{n(n-1)}}\right)}^{\frac{1}{p+q}}},\\ \sqrt{1-{\left(1-{\left(\prod_{\begin{array}{c} i,j=1\\ i\neq j \end{array}}^{n}\left(1-{\left(1-{\left(\tau_{i}^{w_{i}}\right)}^{2}\right)}^{p}\right)\left(1-{\left(1-{\left(\tau_{j}^{w_{j}}\right)}^{2}\right)}^{q}\right)\right)}^{\frac{1}{n(n-1)}}\right)}^{\frac{1}{p+q}}} \end{array}\right) \end{array}\right] \end{array}$$

 □
